# Differential Expression Profile of lncRNAs from Primary Human Hepatocytes Following DEET and Fipronil Exposure

**DOI:** 10.3390/ijms18102104

**Published:** 2017-10-07

**Authors:** Robert D. Mitchell, Andrew D. Wallace, Ernest Hodgson, R. Michael Roe

**Affiliations:** 1 Department of Entomology and Plant Pathology, North Carolina Agromedicine Institute, Campus Box 7647, 3230 Ligon Street, North Carolina State University, Raleigh, NC 27695, USA; rdmitche@ncsu.edu; 2 Toxicology Program, Department of Biology, North Carolina State University, Raleigh, NC 27695, USA; adwallacnc@gmail.com (A.D.W.); ernest_hodgson@ncsu.edu (E.H.); 3 Department of Applied Ecology, Toxicology Program, Department of Biology, North Carolina Agromedicine Institute, North Carolina State University, Raleigh, NC 27695, USA

**Keywords:** DEET, fipronil, long non-coding RNA, lncRNA, primary liver cells, epigenetics, RNA-Seq, transcriptomics, Zika virus, human hepatocytes

## Abstract

While the synthesis and use of new chemical compounds is at an all-time high, the study of their potential impact on human health is quickly falling behind, and new methods are needed to assess their impact. We chose to examine the effects of two common environmental chemicals, the insect repellent *N*,*N*-diethyl-*m*-toluamide (DEET) and the insecticide fluocyanobenpyrazole (fipronil), on transcript levels of long non-protein coding RNAs (lncRNAs) in primary human hepatocytes using a global RNA-Seq approach. While lncRNAs are believed to play a critical role in numerous important biological processes, many still remain uncharacterized, and their functions and modes of action remain largely unclear, especially in relation to environmental chemicals. RNA-Seq showed that 100 µM DEET significantly increased transcript levels for 2 lncRNAs and lowered transcript levels for 18 lncRNAs, while fipronil at 10 µM increased transcript levels for 76 lncRNAs and decreased levels for 193 lncRNAs. A mixture of 100 µM DEET and 10 µM fipronil increased transcript levels for 75 lncRNAs and lowered transcript levels for 258 lncRNAs. This indicates a more-than-additive effect on lncRNA transcript expression when the two chemicals were presented in combination versus each chemical alone. Differentially expressed lncRNA genes were mapped to chromosomes, analyzed by proximity to neighboring protein-coding genes, and functionally characterized via gene ontology and molecular mapping algorithms. While further testing is required to assess the organismal impact of changes in transcript levels, this initial analysis links several of the dysregulated lncRNAs to processes and pathways critical to proper cellular function, such as the innate and adaptive immune response and the p53 signaling pathway.

## 1. Introduction

The study of the impact of environmental chemicals on human health has fallen significantly behind the rate at which we synthesize new chemical compounds. Recent studies suggest that while we generate nearly 10 million new chemical compounds annually, factors such as a lack of funding and reduced public interest and awareness have diminished the amount of research into the potential consequences of human exposure to environmental chemicals [1]. To fully understand the impact of chemicals on human health and provide more rapid and comprehensive methods to evaluate risk, we must take advantage of recent advances in high-throughput DNA sequencing, annotation of the human genome, and bioinformatics, while at the same time using global approaches relating effects of chemical exposure on molecular pathways to whole organism function.

The insect repellent *N*,*N*-diethyl-*m*-toluamide (DEET) and the insecticide fluocyanobenpyrazole (fipronil) are pesticides that have a high potential for human exposure. DEET is applied at a concentration of 5% to 100% to skin to repel insects and other arthropods by approximately 30% of the U.S. population annually [2]. Recently, fetal Zika virus infections in the Americas linked to severe birth defects including microcephaly [3] have further increased our utilization and dependency on DEET. Expectant mothers are encouraged to use this repellent on their skin at a minimum concentration of 30% active ingredient every time they are at risk of being bitten by mosquitoes before and during pregnancy [4]. This level of repetitive use of DEET on human health has never been considered before. Fipronil is an insecticide used to treat companion animals for fleas and ticks as well as around the home for other arthropod control, e.g., termites, roaches, and ants [5]. It is also used as a pesticide in numerous countries around the world to protect crops such as corn and cotton [6,7,8]. However, several countries in the European Union have restricted the use of fipronil citing its potential lethality to honeybees [6]. High doses of fipronil in humans have in some cases resulted in severe vomiting, agitation, and seizures [9].

While both DEET and fipronil have been available commercially for many years (DEET since 1957 and fipronil since 1993), the study of these compounds directly in human systems can improve our understanding of their toxicology [10] and also provide a model for developing a new approach to risk assessment for environmental chemicals in general. Previous studies have measured DEET and fipronil metabolite levels in blood and urine, but no work has been conducted at the DNA/RNA level in relation to human health [11,12]. DEET is metabolized in humans by cytochrome P450 enzymes into the primary metabolites *N,N*-diethyl-*m*-hydroxymethylbenzamide (BALC) and *N*-ethyl-*m*-toluamide (ET). While several P450s have been demonstrated to be active in DEET metabolism, CYP2B6 is the principal P450 responsible for the conversion of DEET to BALC, and CYP2C19 for the conversion of DEET to ET [13]. DEET metabolites are primarily excreted from the human body in urine, but can also be expelled in feces [14]. The predominant metabolite of fipronil is fipronil sulfone (5-amino-1-(2,6-dichloro-4-trifluoromethylphenyl)-3-cyano-4-rifluoromethylsulfonyl-pyrazole), which is primarily metabolized by the cytochrome P450 enzyme CYP3A4 [15,16]. Unlike DEET, fipronil is primarily eliminated in the feces [17]. Our group recently found that exposure of primary human hepatocytes to 100 µM DEET, 10 µM fipronil, and a mixture of 100 µM DEET and 10 µM fipronil significantly altered transcript levels for numerous protein-coding and non-protein coding genes [18]. Here, the goal was to determine what epigenetic elements, i.e., long non-protein coding RNA (lncRNA) transcripts, were significantly differentially expressed after exposure to DEET and fipronil at the same concentrations, and what role they may play in human function.

Long non-protein coding RNAs (lncRNAs) are RNA transcripts greater than 200 nucleotides long which rarely code for protein. They are the largest class of noncoding genes and are processed much like messenger RNA (mRNA), i.e., they are transcribed from active chromatin and can have a 5′ cap and a poly-A tail. The three-dimensional (3-D) structure of lncRNAs predominantly determine what RNA, DNA, or proteins they influence [19]. We now know that protein-coding genes account for less than 2% of the human genome and the majority of transcripts do not code for protein but perform other essential functions [20]. There are several distinct sub-categories of lncRNAs based on their configuration in the genome (i.e., location and proximity to protein-coding genes [21]) that play a critical role at every level of gene regulation [19,22,23]; they serve as transcription signals, transcription factor decoys, guides for chromatin-modifying enzymes, and molecular scaffolds facilitating ribonucleoprotein complex formation [20]. Little is known of their mode(s) of action in general, but even less is known about their function in response to environmental chemicals [24].

Considering an almost complete lack of knowledge of the role of lncRNAs in animal and human responses to environmental chemicals, the objective of this study was to use DEET and fipronil, alone and in a mixture, as models to examine their impact on lncRNA transcript levels in primary human hepatocytes. The research also included an analysis of the potential interaction of lncRNA transcription with that for coding genes to provide leads for future assessments of risks to chemical exposure. Studying DEET and fipronil both alone and in combination in primary liver cells can provide insight on whether processes and pathways are shared or unique among the exposure conditions examined. The use of primary human cells, in general, provides the closest possible estimate of the chemical–human global molecular interaction versus utilization of immortalized cell lines or animal models.

## 2. Results and Discussion

### 2.1. Effects of DEET and Fipronil on LncRNA Versus Protein-Coding Gene Transcription

Primary human hepatocytes were treated with either 100 µM DEET, 10 µM fipronil, or a mixture of 100 µM DEET and 10 µM fipronil. At a significance level of *p* ≤ 0.01, we observed transcripts for 2 lncRNA genes that were upregulated and 18 that were downregulated by 100 µM DEET. This accounted for 0.04% of the total number of coding and noncoding genes identified in the latest Ensembl release (Ensembl release 87) of the annotated human genome [25]. Specifically, of all of the annotated categories recognized by the Ensembl project that include coding genes, noncoding genes (defined as small noncoding genes, long noncoding genes, and miscellaneous noncoding genes), and pseudogenes, 20 of the 56,384 genes were lncRNA genes whose transcripts were differentially expressed after exposure to 100 µM DEET. When primary human hepatocytes were treated with 10 µM fipronil, there were 76 lncRNA genes whose transcripts were upregulated and 193 downregulated, accounting for 0.48% of the total number of coding and noncoding genes identified in the latest human genome annotation (i.e., 269 of 56,384 genes). When primary human hepatocytes were treated with a mixture of 100 µM DEET and 10 µM fipronil, we observed 75 lncRNA genes whose transcripts were upregulated and 258 downregulated. This accounted for 0.59% of the total number of coding and noncoding genes identified in the latest human genome annotation (i.e., 333 of 56,384 genes). We observed a more-than-additive effect as the sum of the lncRNAs dysregulated by the 100 µM DEET treatment and the 10 µM fipronil treatment was 289, but the two chemicals together elicited up- or downregulation of 333 lncRNAs. We define “more-than-additive” as the number of dysregulated transcripts when cells were treated with DEET and fipronil together that was greater than the number of dysregulated transcripts when cells were treated separately with each compound with the concentration of each compound remaining the same in all treatments. This definition does not include a detailed dose–response evaluation, but is simply meant as a statement describing a mathematical calculation.

In this study, we included transcribed pseudogenes as lncRNAs and specifically defined an lncRNA as any non-protein coding gene whose transcripts were ≥200 nucleotides long. A pseudogene is a highly similar copy of a protein-coding gene. A protein-coding gene that is similar to a specific pseudogene is termed a parental gene to that pseudogene and no longer produces a functional protein product in most cases [26]. Pseudogenes typically regulate parental genes as lncRNA transcripts, and previous studies have established that pseudogenes, when transcribed, function as drivers of gene regulation like that for other lncRNAs. Not all pseudogenes are actively transcribed (although the ones identified in this study were transcribed); some estimate that only 2–20% of pseudogenes in the human genome are actively transcribed [26,27,28]. Pseudogenes function as regulators of target genes when their transcripts interact with target gene promoters and are processed into short noncoding RNAs that hybridize to the protein-coding sense strand transcripts [28]. Experimental evidence supports the role of transcribed pseudogenes as regulating messenger RNAs (mRNAs) via small interfering RNAs [29], regulating other lncRNA transcripts [30], and functioning as microRNA (miRNA) decoys [31].

One of the two lncRNA genes whose transcripts were upregulated by 100 µM DEET was a pseudogene, and 5 of the 18 (28%) downregulated transcripts were pseudogenes. Thirty-four of the 76 (45%) lncRNA genes whose transcripts were upregulated by 10 µM fipronil were pseudogenes, and 72 of the 193 (37%) downregulated transcripts were pseudogenes. Thirty-two of the 75 (43%) lncRNA genes whose transcripts were upregulated by the 100 µM DEET plus 10 µM fipronil mixture were pseudogenes, and 97 of the 258 (38%) downregulated transcripts were pseudogenes. Therefore, approximately 40% of the dysregulated lncRNAs in each treatment were pseudogenes. These findings indicate that many genes that once coded for active proteins, but were thought to currently be inactive (pseudogenes, also known as lncRNAs), may still play a prominent role in gene regulation. This may have arisen from the accumulation of multiple mutations over time, rendering a protein-coding gene inactive. Then, these lncRNAs were “repurposed” to influence the activity of other noncoding and coding elements without coding for proteins themselves. However, our understanding of how lncRNA transcripts interact amongst themselves, other epigenetic elements, protein-coding genes, and gene products is still in its infancy.

**Figure 1:**
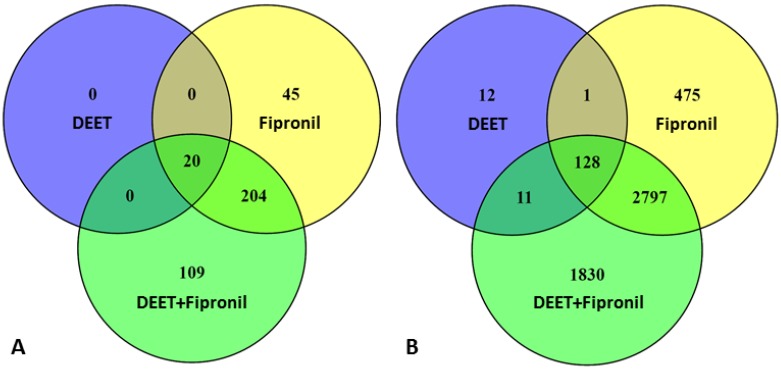
Relationships between the number of long non-protein coding RNAs (lncRNAs) whose transcripts were significantly (*p* ≤ 0.01) differentially up- and downregulated (**A**) and protein-coding genes whose transcripts were significantly (*p* ≤ 0.01) differentially expressed (**B**) when primary human hepatocytes were treated with DEET (100 µM), fipronil (10 µM), or a mixture of the two (100 µM DEET and 10 µM fipronil) for 72 h.

Figure 1A shows that 20 of the lncRNAs whose transcripts were significantly differentially expressed (*p* ≤ 0.01) were shared among all three treatment conditions (100 µM DEET, 10 µM fipronil, and 100 µM DEET combined with 10 µM fipronil). Interestingly, there were no lncRNAs whose differentially expressed transcripts were unique only to the 100 µM DEET treatment. There were 204 lncRNA genes whose transcripts were differentially expressed that were shared between the 10 µM fipronil treatment and the 100 µM DEET plus 10 µM fipronil treatment, 45 that were unique to the fipronil-only treatment, and 109 that were unique to the DEET plus fipronil mixture. Figure 1B shows the relationship between the statistically significant up- and downregulated protein-coding genes at *p* ≤ 0.01, which we defined as any genes that were processed into messenger RNA (mRNA) and deemed protein-coding via the National Center for Biotechnology Information (NCBI) database [32]. This definition excludes any noncoding RNAs or the few pseudogenes that are now thought to code for protein and may be reclassified in the near future [33].

Table 1 shows the 20 lncRNAs whose transcripts were differentially expressed across all three treatment conditions. This group of affected lncRNAs was interesting because the chemistry and mode of action of DEET and fipronil are completely different and yet the same lncRNA transcripts were affected. Of the 20, only 5 lncRNAs (25%) had assigned Gene Ontology (GO) terms at the biological process, cellular component, or molecular function level. These will be discussed in more detail below, but it is worth noting that the log2 fold change (log2FC), a common metric for differential expression values [34], was very similar and in the same direction, either up- or downregulated, for each lncRNA among the 100 µM DEET, 10 µM fipronil, and 100 µM DEET plus 10 µM fipronil conditions. While all 20 examples in Table 1 were up- or downregulated in the same manner, this terminology differs from the more-than-additive effect we observed. The “more-than-additive” effect refers to a generalization and did not pertain to every single transcript, but rather the overall number of genes whose transcripts were up- or downregulated under a specific treatment condition. Table S3 provides additional information about these 20 lncRNAs that were dysregulated across all three treatment conditions, including chromosomal coordinates and gene accession numbers.

### 2.2. Chromosomal Mapping of Dysregulated LncRNAs

**Figure 2:**
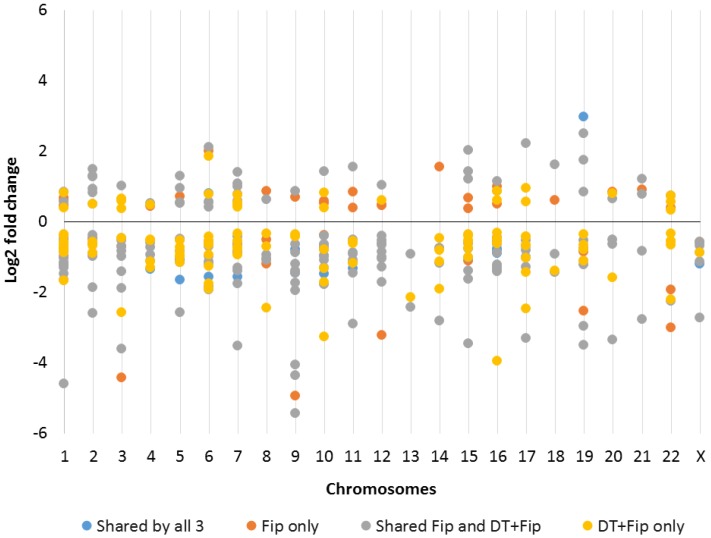
Log2 fold change of transcripts significantly differentially expressed from long non-protein coding RNA genes by chromosome (*p* ≤ 0.01). Shared by all 3 means those transcripts were differentially expressed in all three treatments; Fip only means those transcripts were only differentially expressed when hepatocytes treated with 10 µM fipronil; Shared Fip and DT+Fip means those transcripts were only differentially expressed when hepatocytes treated with fipronil or a combination of DEET and fipronil, but not 100 µM DEET alone; and DT+Fip only means those transcripts were only differentially expressed with the combination of DEET and fipronil, but not each treatment alone. A single representative log2 fold change value was used for transcripts that were differentially expressed under more than one treatment condition. * A representative log2 fold change refers to the average log2 fold change for any genes whose transcript expression was affected by more than one treatment, like shared by all 3, where the same genes were dysregulated by all three treatment conditions.

RNA-Seq (transcriptomic) data were analyzed to reveal the identity and genomic location of lncRNAs relative to protein-coding genes whose transcripts were significantly differentially expressed (*p* ≤ 0.01) after exposure to 100 µM DEET, 10 µM fipronil, and a mixture of 100 µM DEET with 10 µM fipronil. Figure 2 shows the distribution and magnitude (in ± log2FC values) of all differentially expressed lncRNA transcripts across all human chromosomes. Every human chromosome was affected in some way by one or more of the test treatments, and the magnitude of up- or downregulation of dysregulated lncRNA gene transcripts was typically similar across treatments as well.

**Figure 3:**
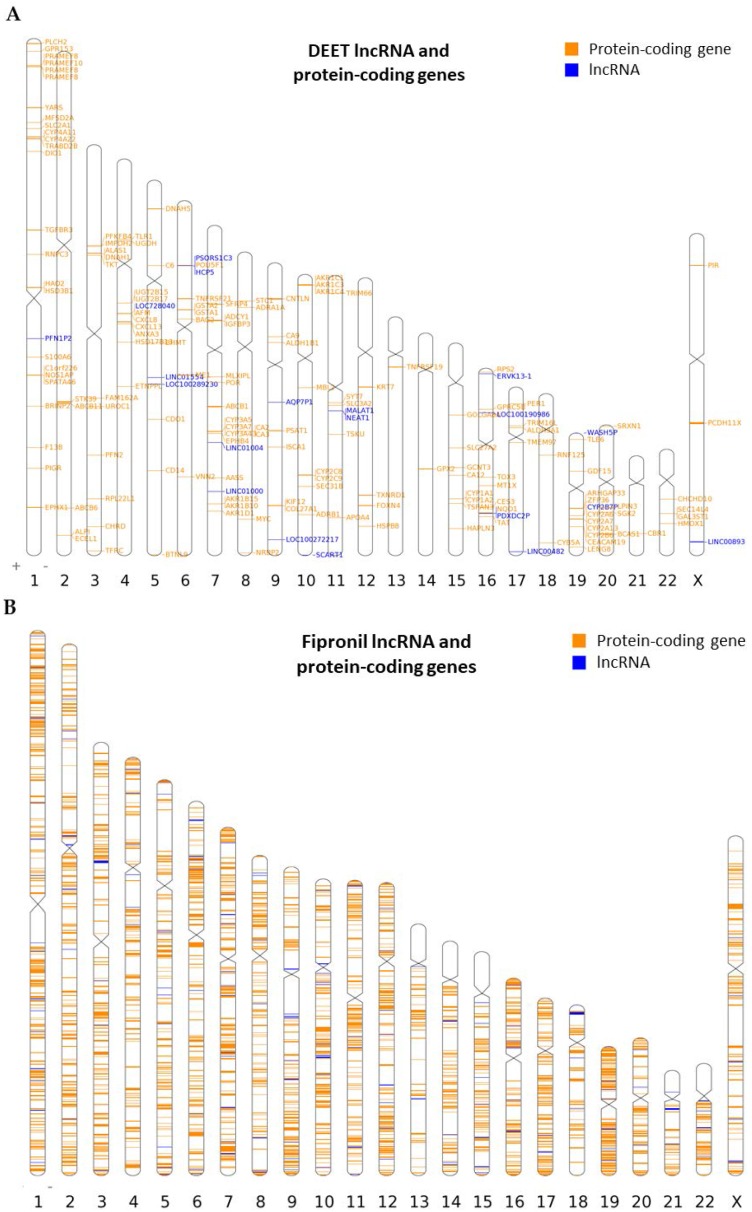

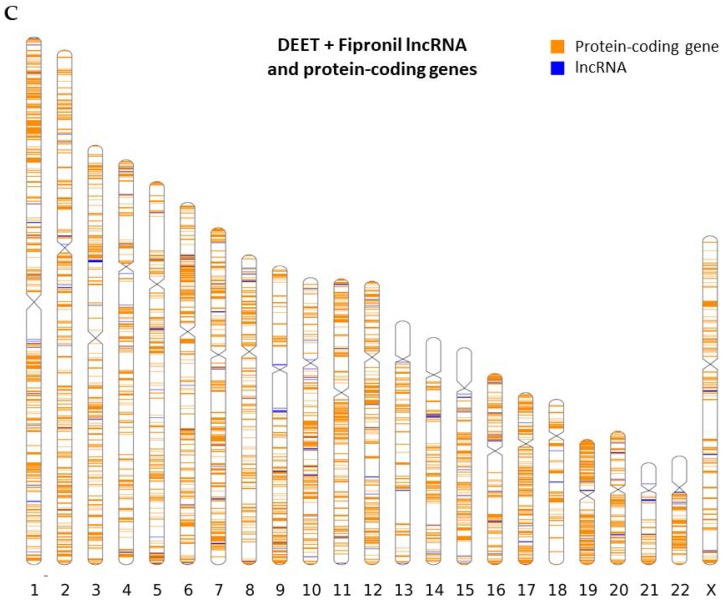
Chromosome maps showing location of lncRNAs with differentially expressed transcripts in relation to dysregulated protein-coding genes after exposure of primary human hepatocytes to 100 µM DEET, 10 µM fipronil, or a mixture of 100 µM DEET and 10 µM fipronil. (**A**) Chromosomal location of lncRNAs and protein-coding genes significantly dysregulated (*p* ≤ 0.01) when hepatocytes exposed to 100 µM DEET; (**B**) chromosomal location of lncRNAs and protein-coding genes significantly dysregulated (*p* ≤ 0.01) when hepatocytes exposed to 10 µM fipronil; (**C**) chromosomal location of lncRNAs and protein-coding genes significantly dysregulated (*p* ≤ 0.01) when hepatocytes exposed to a mixture of 100 µM DEET and 10 µM fipronil. For (**A**), blue bars and gene symbols denote identity and location of lncRNAs with differentially expressed transcripts (both up- and downregulated). Orange bars and gene symbols denote identity and location of lncRNAs with differentially expressed protein-coding gene transcripts (both up- and downregulated). For (**B**,**C**), blue bars denote location of lncRNAs with differentially expressed transcripts (both up- and downregulated). Orange bars denote location of protein-coding genes with differentially expressed transcripts (both up- and downregulated).

Genomic location data were analyzed with the Idiographica algorithm [36] to visualize the location and orientation of dysregulated lncRNA genes in relation to dysregulated protein-coding genes across all human chromosomes. There were five instances where a dysregulated lncRNA (from the 100 µM DEET treatment) occupied genomic space within 1000 kilobases (kb) of a dysregulated protein-coding gene transcription start site (TSS). This 1000 kb range from the center of an lncRNA gene to a neighboring protein-coding gene TSS constituted a regulatory region as defined by the Genomic Regions Enrichment of Annotations Tool (GREAT) [37]; this will be discussed in more detail later. A TSS is the first nucleotide base in a section of DNA where RNA polymerase II begins to synthesize a complementary RNA transcript at the 5’ end of a gene [38]. In the 100 µM DEET treatment, there were even two examples of a dysregulated lncRNA gene residing within 300 kb of another dysregulated lncRNA gene, suggesting that some of these long noncoding elements may actually influence the activity of one another and/or the protein-coding genes with which each interacts. Figure 3A shows the chromosomal distribution of the dysregulated lncRNAs (20 genes; *p* ≤ 0.01) and dysregulated protein-coding genes (152 genes, *p* ≤ 0.01) together after 100 µM DEET treatment. Chromosomes 1, 4, 5, 6, 7, 9, 11, 16, 17, 19, and X had lncRNAs that were dysregulated by the treatment, and we could clearly visualize (via the ideogram) the protein-coding genes that were closely oriented with those 20 lncRNA genes. Figure 3B–C shows the lncRNA genes and protein-coding genes whose transcripts were differentially expressed when primary human hepatocytes were treated with 10 µM fipronil or a mixture of 100 µM DEET plus 10 µM fipronil, respectively. There were too many genes listed to label gene symbols, so the dysregulated lncRNA genes are simply labeled with blue bars and the dysregulated protein-coding genes are labeled with orange bars. All gene markers on the chromosomal maps represent their relative size (in base pairs) and position in relation to size and orientation to all the known coding and noncoding genes on each human chromosome.

A chromosome-by-chromosome analysis was conducted in an attempt to determine if any correlation existed between the distribution of dysregulated lncRNAs and dysregulated protein-coding genes. The findings are reported in Supplementary Tables S1 and S2. Supplementary Sections S1 and S2 list the chromosomes with the highest and lowest number of lncRNAs and protein-coding genes that were dysregulated by all three of our treatments. We found no clear correlation for each chromosome between the highest percentages of dysregulated lncRNAs versus chromosomes with the highest percentage of dysregulated coding genes. However, there are several instances of dysregulated lncRNAs occurring within a regulatory region of a dysregulated protein-coding gene, some of which will be displayed below.

### 2.3. Association of LncRNA Genes with Protein-Coding Genes

To identify which protein-coding genes were associated (i.e., within a pre-determined distance and with a potentially functional relationship) with lncRNAs whose transcripts were differentially expressed after the 100 µM DEET treatment, the 10 µM fipronil treatment, and the 100 µM DEET plus 10 µM fipronil treatment, we used the Genomic Regions Enrichment of Annotations Tool (GREAT). Neighboring protein-coding genes were defined by the GREAT algorithm as those within 1000 kb of an input genomic region (lncRNAs we found to be dysregulated by our treatments in this case). The GREAT algorithm assumed that lncRNAs within 1000 kb of a neighboring gene transcription start site (TSS) could affect the transcription of that neighboring gene, thereby assigning putative functionality to the input lncRNA. The program only considered neighboring protein-coding genes and did not associate lncRNA genes with other noncoding gene transcription regions [37]. Supplementary Figure S1 shows the readings obtained when the dysregulated lncRNAs for each of our three treatments were interrogated with the GREAT algorithm for their proximity (within 1000 kb) to protein-coding genes of known function. A short explanation of the readings is discussed in Supplementary Section S3.

Table 2 shows the 20 lncRNAs whose transcripts were differentially expressed after 100 µM DEET exposure and their neighboring protein-coding genes and lncRNA genes. Included in Table 2 are the protein-coding genes that were dysregulated (shown by an asterisk) and have been described before [18]. Supplementary Table S3 elaborates on information about the specific lncRNAs presented in Table 2. Supplementary Table S3 provides the GenBank accession number, chromosome coordinates, and whether the lncRNA was significantly up- or downregulated after treatment for each of the 20 dysregulated lncRNAs. Supplementary Table S4 shows the same information as Supplementary Table S3 for the 10 µM fipronil treatment, and Supplementary Table S5 shows the same from the 100 µM DEET plus 10 µM fipronil treatment. After GREAT analysis was utilized to establish the identity of protein-coding genes that were associated (within 1000 kb) with the dysregulated lncRNAs from each treatment condition; we used these genes as input into a functional analysis algorithm to determine within what biological processes and pathways these genes may be active. The findings of the functional characterization are presented later in Section 2.4.

**Figure 4:**
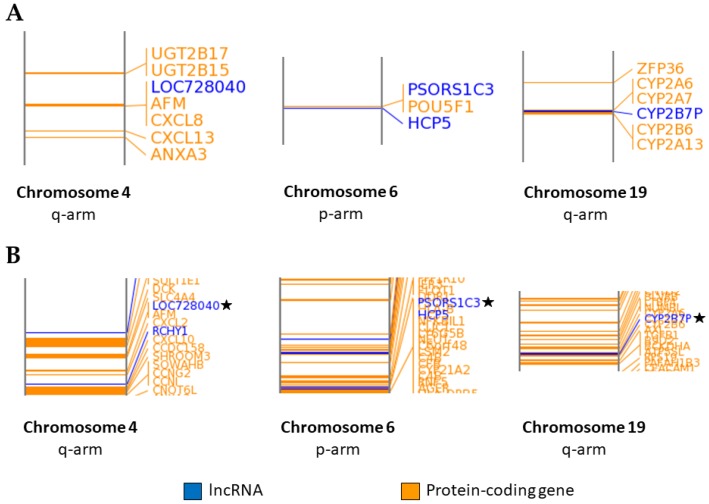
Chromosomal location of lncRNA genes within 1000 kb of protein-coding genes with differentially expressed transcripts after primary human hepatocytes were exposed to 100 µM DEET or 10 µM fipronil. (**A**) Location of dysregulated lncRNAs and neighboring (within 1000 kb) protein-coding genes affected by 100 µM DEET on selected chromosomes and (**B**) location of dysregulated lncRNAs and neighboring protein-coding genes affected by 10 µM fipronil on selected chromosomes. p-arm = short arm of chromosome; q-arm = long arm of chromosome; black star = corresponding lncRNA from 10 µM DEET treatment.

Figure 4A displays magnified regions on specific chromosomes (via the Idiographica algorithm) where lncRNAs with differentially expressed transcripts were closely associated (based on distance in base pairs) with neighboring dysregulated protein-coding genes for the 100 µM DEET treatment. The three specific regions selected are examples from a total of five cases of dysregulated lncRNAs that were within 1000 kb of protein-coding genes that were dysregulated by exposure to 100 µM DEET. Figure 4B shows the dysregulated lncRNAs and protein-coding genes from the 10 µM fipronil treatment in the same regions that were highlighted with the DEET-only data in Figure 4A. The same dysregulated protein-coding and lncRNA genes that were neighbors in the 100 µM DEET treatment were visible for the fipronil treatment in addition to many other protein-coding genes that were not dysregulated by the 100 µM DEET treatment; recall the fipronil treatment activated 13.5X as many differentially expressed lncRNA gene transcripts and 21.3X as many dysregulated protein-coding gene transcripts as the DEET-only treatment. The analysis presented in the following sections includes the coding and noncoding genes that were within 1000 kb of neighboring lncRNAs (termed *cis* activation) because the GREAT algorithm that we used did not focus on long-distance relationships (*trans* activation). It is known that certain lncRNAs can affect distant coding and noncoding genomic regions, but these associations have been poorly studied and are less understood in comparison to *cis* activation; for this reason, we chose to only focus on the closer proximity relationships [39].

### 2.4. Functional Characterization of Dysregulated LncRNAs

**Figure 5:**
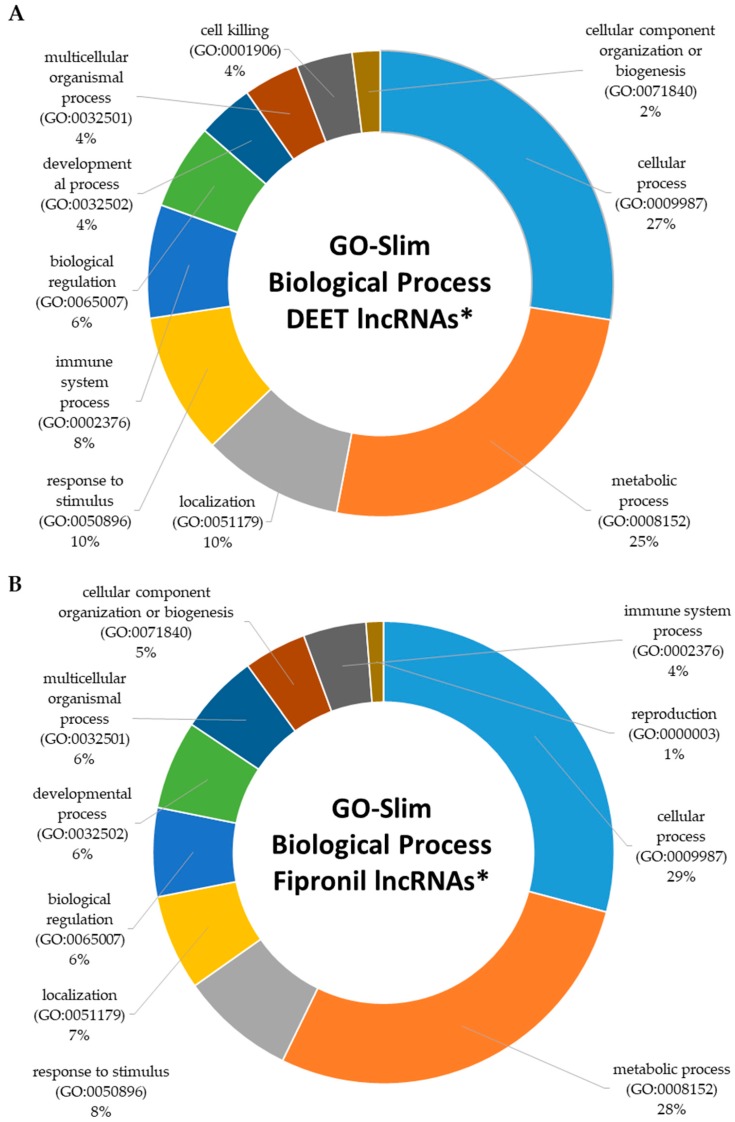

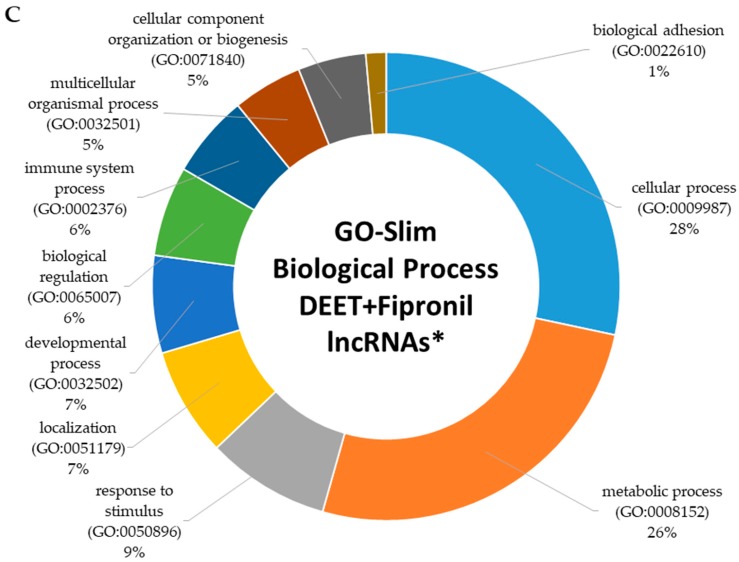
Top 10 biological processes affected by exposure of primary human hepatocytes to (**A**) 100 µM DEET; (**B**) 10 µM fipronil; and (**C**) 100 µM DEET plus 10 µM fipronil. * Assignments were made using GO-Slim analysis of dysregulated lncRNAs and “associated” protein-coding genes (as determined using the GREAT algorithm as described in methods section). GO = gene ontology assignment number.

The Protein ANalysis THrough Evolutionary Relationships (PANTHER) classification system [40] was utilized to classify the lncRNA and protein-coding genes that were linked to one another in the GREAT analysis into functional groups and pathways. PANTHER GO-slim annotation focusing on biological processes showed that components of 11 biological processes were activated by 100 µM DEET. Figure 5A displays the top 10 of these 11 biological processes whose lncRNA and neighboring protein-coding gene transcripts were differentially expressed by 100 µM DEET (based on the number of genes included in each process). The two highest represented were cellular processes (27%) and metabolic processes (25%), but several other critical biological processes, such as immune system processes, cell killing, and biological regulation, were also included. Supplementary Table S6 displays all 11 biological processes whose dysregulated lncRNAs and neighboring coding genes were associated. A search of the approximately 177 primary signaling pathways in the PANTHER database found that several of our dysregulated lncRNAs and neighboring protein-coding genes from the DEET-only treatment were associated with Ras pathway activity, the PI3 kinase pathway, the p53 pathway, and immune response pathways among others. This is discussed in more detail later.

When we input our list of dysregulated lncRNAs and associated protein-coding genes from the 10 µM fipronil treatment, we obtained matches for 14 biological processes, 11 of which were included in the 100 µM DEET analysis (Figure 5B). The 3 processes that were not included in the DEET-only dataset were reproduction, biological adhesion, and rhythmic processes (Supplementary Table S7 provides all 14 biological processes). For the 100 µM DEET plus 10 µM fipronil mixture, we found the same 14 biological processes activated that were also elicited by the 10 µM fipronil treatment, of which the top 10 are shown in Figure 5C and a complete list can be found in Supplementary Table S8.

### 2.5. Metabolic Signaling Pathways Associated with Dysregulated LncRNAs

**Figure 6:**
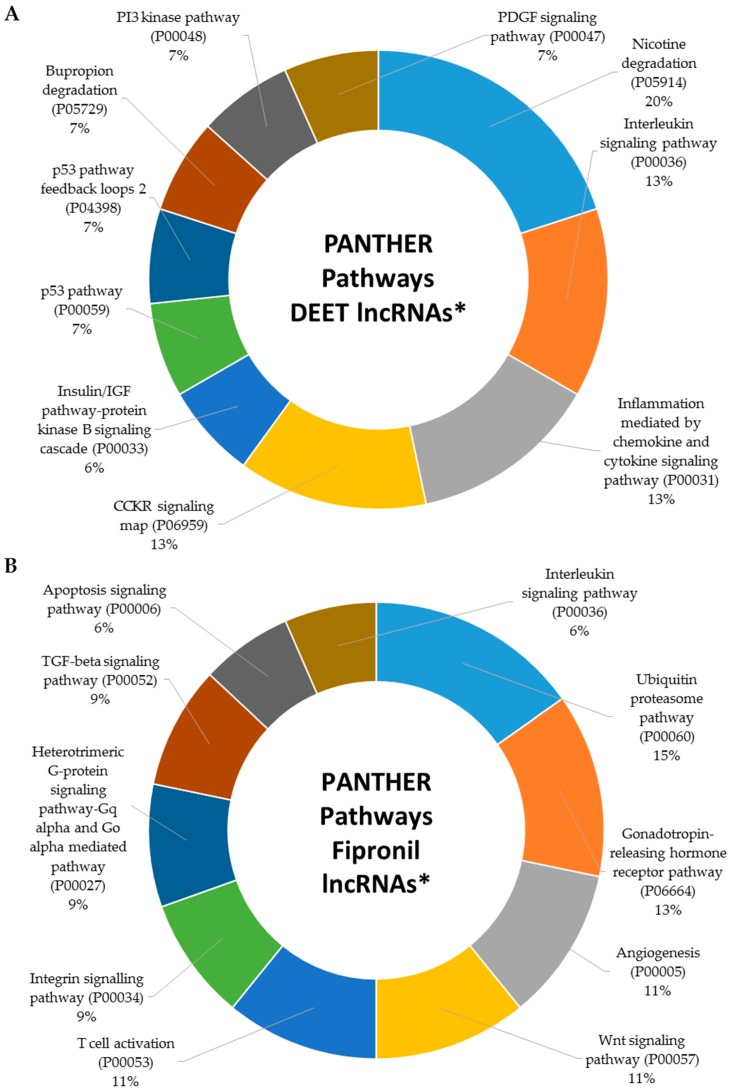

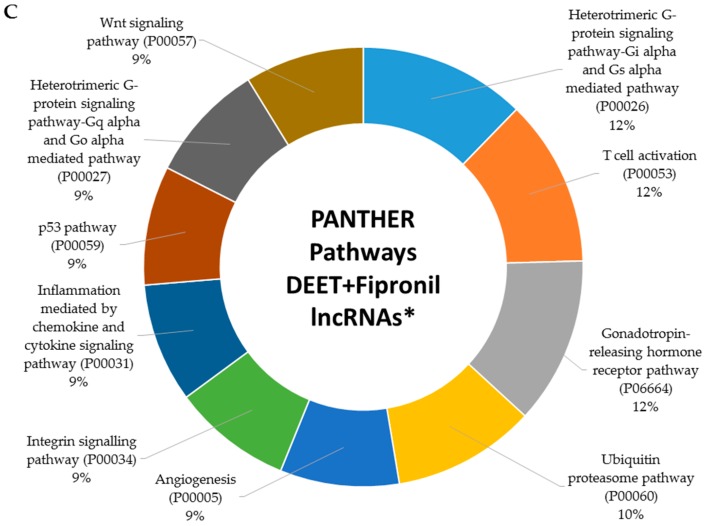
Top 10 signaling pathways affected by exposure of primary human hepatocytes to (**A**) 100 µM DEET; (**B**) 10 µM fipronil; and (**C**) 100 µM DEET plus 10 µM fipronil. * Assignments were made using PANTHER analysis of dysregulated lncRNAs and “associated” protein-coding genes (as determined using the GREAT algorithm as described in the Methods section). Letters and numbers in parenthesis are PANTHER identifiers.

In our previous work, we established that several protein-coding genes whose transcription was up- and downregulated by DEET and fipronil exposure were involved in critical metabolic signaling pathways [18]. Here, we establish that DEET and fipronil influenced the transcription of many lncRNAs that were either directly or indirectly involved in the activity of signaling pathways key to normal cellular function. Figure 6A displays the top 10 signaling pathways (based on the number of genes included in each pathway) affected by exposure to 100 µM DEET. The 11th signaling pathway affected by 100 µM DEET that is not displayed in Figure 6A is the Ras pathway. Figure 6B–C displays the top 10 signaling pathways affected by 10 µM fipronil and a mixture of 100 µM DEET and 10 µM fipronil, respectively. In total, the 10 µM fipronil treatment affected 45 signaling pathways (Supplementary Table S9) and the mixture of 100 µM DEET and 10 µM fipronil affected 68 signaling pathways (Supplementary Table S10).

When we searched the signaling pathway database with the 10 µM fipronil data, we obtained 45 matches that included all 11 pathways affected by the 100 µM DEET treatment. This was expected, since all of the dysregulated lncRNAs found in the 100 µM DEET treatment were also affected by the 10 µM fipronil treatment. However, the fipronil-only treatment activated 34 additional signaling pathways. The mixture of 100 µM DEET and 10 µM fipronil activated many more signaling pathways than either DEET or fipronil alone. We found 68 total signaling pathways associated with the mixture, of which 11 were shared with the 100 µM DEET and 10 µM fipronil treatments and the 34 additional matches from the fipronil-only treatment were shared, leaving 23 pathways unique to the response of primary human hepatocytes to a mixture of 100 µM DEET and 10 µM fipronil. Some of the pathways most affected by all three treatments included the immune system, p53, Ras, and Wnt signaling pathways. Each is critical to normal cellular function, and interference in these processes is directly linked to disease presentation and progression.

There is much cross talk between metabolic signaling and the immune system or the p53 signaling pathway. Initiation of a targeted immune response, lymphocyte activation for example, is bioenergetically expensive. Therefore, precise control of the interplay between metabolic signaling with the immune system (or other signaling processes we identified as dysregulated in this work) is essential. Interaction between these complex systems is now a major focus in the study of metabolic disorders and cancer [41,42]. The p53 tumor suppressor gene regulates critical metabolic changes in cells. It carefully balances glycolysis and oxidative phosphorylation as well as the autophagy pathway; dysregulation of the p53 pathway can have a profound influence on the behavior of normal and diseased cells [43,44].

#### 2.5.1. Dysregulated LncRNAs Involved in Innate and Adaptive Immunity

The immune system is a defense mechanism that protects the body from foreign invaders, such as bacteria and viruses. Deficiencies in this network can result in autoimmune diseases, inflammatory diseases, and cancer. There is great concern nearly tantamount to a crisis situation regarding the current emergence of drug-resistant bacteria, of which our bodies can no longer successfully combat via both innate and adaptive immunity [45]. Environmental chemicals that further weaken the immune system exacerbate an already dire dilemma. Transcripts of lncRNA NEAT1 were significantly downregulated in all three treatments (see Table 1), and are known to influence immune gene expression and immune cell functions. NEAT1 does this by binding to splicing factor proline and glutamine rich (SFPQ), which in turn activates the transcription of the gene that codes for chemokine interleukin 8 or IL8 [19]. We did not see differential expression in *SFPQ*, but we did observe the upregulation of *IL8* in the 100 µM DEET and 100 µM DEET plus 10 µM fipronil mixture but not in the 10 µM fipronil condition. The lncRNA growth arrest-specific transcript 5 (GAS5), whose transcripts were upregulated in the 10 µM fipronil and 100 µM DEET plus 10 µM fipronil mixture treatments, is critical in regulation of the cell cycle and apoptotic control of T cells, and its normal expression is linked to tumor suppression [46]. However, GAS5 overexpression has been shown to inhibit the growth of T cells and promote spontaneous apoptosis [47]. Noncoding repressor of NFAT (NRON) is known to inhibit nuclear factor of activated T cells (NFAT). While we did not observe dysregulation of *NRON*, we did see significant downregulation of *NFAT5* transcripts in both the fipronil-only and DEET plus fipronil mixture treatments [19].

Using GO-Slim annotations in PANTHER, we identified four additional genes neighboring (within 1000 kb) lncRNAs that were involved in either antigen processing and presentation or the immune response in the 100 µM DEET treatment. MHC class I polypeptide-related sequence A and B (*MICA* and *MICB*) were two of these genes neighboring the lncRNA HLA Complex P5 gene (*HCP5*). Products of the C-X-C Motif Chemokine Ligand 8 gene (*CXCL8*), also associated with *HCP5*, and the Major Histocompatibility Complex, Class I, C protein gene (*HLA-C*) were identified as immune response components. *HLA-C* is associated with the dysregulated lncRNAs psoriasis susceptibility 1 candidate 3 (*PSORS1C3*) and *HCP5*. *HCP5* is an endogenous retrovirus that has become part of the human genome, and is specifically associated with HIV-1 viral load where the expression of one variant of the final protein product of *HCP5* is shown to interact with HIV-1 and reduce its viral presence [48]. When we expanded our signaling pathway search to the dysregulated lncRNAs and neighboring protein-coding genes from the 10 µM fipronil treatment we obtained matches for 29 immune system components, and if we expand to the 100 µM DEET plus 10 µM fipronil lncRNAs and associated protein-coding genes we obtain 45 matches to immune functions. There were several dysregulated genes from our previous study that were included, but which the current study was not limited to, since they also function within the immune response pathway (*FOS*, *JUNB*, *LCP2*, *TLR1*, *TLR2*, *TLR3*, and *TLR4*). Serine/threonine-protein kinase B-Raf (BRAF), whose gene neighbors the differentially expressed lncRNA *NDUFB-AS1* (whose transcripts were downregulated by both the 10 µM fipronil and 100 µM DEET plus 10 µM fipronil treatments), is a protein that transmits signals from the outside to the inside of a cell and is ultimately involved in cell growth and proliferation. Mutations in this gene have been shown to lead to cancer, since the accumulation of mutations in *BRAF*, like many other genes critical to normal human processes, contributes to the development of cancer [49]. The Hallmarks of Cancer, published in 2000 (updated in 2011), was a seminal paper that established six cellular alterations necessary to dictate malignant growth that we still follow today [50,51]. These alterations can arise from perturbations of components in any of the key metabolic processes discussed here.

#### 2.5.2. Dysregulated LncRNAs Involved in the Transformation-Related Protein 53 (p53) Signaling Pathway

The p53 pathway helps the body to respond to stress and prevents genome mutations by activating cell cycle arrest, cellular senescence, DNA repair, or apoptosis. Transformation-related protein 53 (p53) is any isoform of the protein coded from the *trp53* gene and has been referred to as the “guardian of the genome” largely due to its function as a tumor suppressor gene [52]. While p53 regulates a large set of genes, the p53 pathway is itself under the control of multiple self-regulatory pathways, including seven negative and three positive feedback loops [53]. Several lncRNAs have already been implicated in regulation of the p53 pathway at various levels, and we found that some previously described lncRNAs and neighboring protein-coding genes were affected by all three of our treatment conditions in some capacity. The lncRNA genes *MALAT1* and *MEG3*, whose transcripts are termed p53 regulators, were dysregulated by both DEET and fipronil as described previously. The lncRNA *H19*, whose transcripts are considered p53 effectors, was also dysregulated [20]. We identified one protein-coding gene neighboring the dysregulated lncRNA gene *ERVK13-1* that was connected with p53 activity in the 100 µM DEET treatment called *PDPK1*. In the 10 µM fipronil and 100 µM DEET plus 10 µM fipronil treatments, there were five (*MDM2*, *RCHY1*, *PDPK1*, *CDKN2B*, and *PRKAB2*) and seven (*PIK3C3*, *HDAC2*, *MDM2*, *RCHY1*, *PDPK1*, *CDKN2B*, and *PRKAB2*) lncRNA genes or lncRNA-associated protein-coding genes linked to p53 activity, one of which was mouse double minute 2 homolog (*MDM2*). The MDM2 protein, associated with the dysregulated lncRNA gene *LOC100130075*, is a well-known regulator of the p53 pathway as it controls six of the ten known feedback loops mentioned above. The presence of MDM2 limits the growth-suppressive functions of p53 by degrading the protein, and MDM2 levels decrease when p53 must respond to stress [54]. In this study, *LOC100130075* transcripts were downregulated by the 10 µM fipronil and 100 µM DEET plus 10 µM fipronil treatments, which could have affected MDM2 activity in relation to p53. The protein 3-phosphoinositide-dependent protein kinase 1 (PDPK1), whose TSS neighbors the lncRNA gene *ERVK13-1* whose transcripts were downregulated in all three treatment conditions, is also connected with p53 activity. PDPK1 is called the “master kinase” because of its importance in signaling pathways tied to growth factors, hormones, and insulin [55]. PDPK1 is a negative regulator of p53, and its levels are elevated in several different types of cancer, including prostate, liver, and breast cancer. Its inhibition has been shown to hinder tumor growth, and it is a promising candidate for cancer intervention [56]. We also noted that several of the protein-coding transcripts that we identified as significantly up- or downregulated previously were directly or indirectly related to p53 functionality, including *ATM*, *SIRT1*, *CDKN2A*, *CDKN2B*, *CREBBP*, *PAK2*, *PDRG1*, *TP53INP2*, *CDIP1*, *PERP*, *RRM2B*, *TRIAP1*, *CSNK2A1*, *CSNK2A2*, and *HIPK2,* which implies that the lncRNAs linked to p53 activity whose transcripts were up- or downregulated by our treatments could have had an influence on these genes or their protein products as well, since they all play some role in the same molecular pathway.

### 2.6. Specific Well-Studied LncRNAs Dysregulated by DEET and Fipronil

We identified several genes (coding and noncoding) that were significantly affected (*p* ≤ 0.01) by 100 µM DEET, 10 µM fipronil, and a mixture of 100 µM DEET plus 10 µM fipronil. Many of those genes were linked to critical biological processes and signaling pathways as described previously. In Supplementary Section S4, we discuss a subset of lncRNAs whose transcript expression was affected in our experiments that have been well-studied in the field; these are focused on in more detail than other known lncRNAs, and a summary of their expression across our three treatment categories is displayed in Table 3.

## 3. Materials and Methods

### 3.1. Primary Human Hepatocytes

Plated primary human hepatocytes were obtained from Life Technologies Corporation (Carlsbad, CA, USA). Upon arrival, the medium the cells were shipped in was removed and replaced with fresh, sterile William’s E Medium supplemented with additives necessary to properly maintain primary human hepatocytes in culture as previously reported [15,18,57]. The plated cells were then placed in a humidified incubator (relative humidity of 95%) at 5% CO_2_/95% air at a temperature of 37 °C for a total of 48 h (media replaced once after 24 h to assess viability and quality).

### 3.2. DEET and Fipronil Treatments

Treatments with DEET and fipronil began 48 h after the cells arrived. On the same plate, three wells of primary human hepatocytes were each inoculated with DEET (purity >98%; Cat. No. F2284, Chem Service, Inc., West Chester, PA, USA; final concentration 100 µM in each well), fipronil (purity >98%; Cat. No. PS2136, Chem Service, Inc., West Chester, PA, USA; 10 µM), DEET and fipronil (mixed together before adding; 100 µM DEET and 10 µM fipronil), and DMSO. The insecticides were added to the culture media dissolved (*wt*/*vol*) in DMSO (dimethyl sulfoxide; ≥99.7% pure; Cat. No. BP231-100, Fisher Scientific International, Inc., Hampton, NH, USA). The amount of DMSO (0.1% final concentration) was the same for all treatments, and was previously shown to produce minimal cytotoxicity or changes in gene expression for hepatocytes in culture (LeCluyse et al. [58]).

The concentration levels chosen for the insecticides were determined from the dose–response data for DEET and fipronil, respectively, from Das et al. [15,57] using the same experimental assay conditions for primary human hepatocytes. The DEET concentration chosen (100 µM) is in the intoxication range of what would be expected in human blood within 8 hours after a dermal treatment, 6.7-fold greater than what would be expected in human blood when DEET is appropriately applied at a maximum dose, and approximately one-fifth that for a person subjected to an acute intentional oral overdose of DEET [59]. No data are available on fipronil levels in human blood; the fipronil treatment level in this study was one-tenth that of DEET. Once all cells received the treatment or carrier only, they were incubated undisturbed for 72 h in a humidified incubator under the environmental conditions previously described.

### 3.3. RNA Isolation and Quality Assessment

After 72 h of treatment, hepatocytes were washed three times with 1X phosphate-buffered saline (PBS, Cat. No. 10010-023, Life Technologies, Carlsbad, CA, USA) and harvested from each individual well (three DEET wells, three fipronil wells, three DEET plus fipronil wells, and three control wells) in a lysis buffer suspension. The suspension was stored at −80 °C or processed immediately via the RNeasy Mini Kit (Cat. No. 74104, Qiagen Inc., Valencia, CA, USA) RNA isolation protocol. Each isolated total RNA sample was separately analyzed for purity on an Agilent 2100 Bioanalyzer (Agilent Technologies, Santa Clara, CA, USA) by the North Carolina State Genome Sciences Laboratory (Raleigh, NC, USA). No samples with an RNA Integrity Number (RIN) of less than 9.0 were used for sequencing; the lowest RIN obtained was 9.4.

### 3.4. Illumina Sequencing

Sequencing of all treatments and the controls was performed on the Illumina HiSeq 2000 platform (Illumina, Inc., San Diego, CA, USA) at the Beijing Genomics Institute collaborative genome center at the Children’s Hospital of Philadelphia (BGI@CHOP, Philadelphia, PA, USA) and has been described before [18].

### 3.5. RNA-Seq Analysis

Elements of the Tuxedo suite pipeline [60] were used to analyze the RNA-Seq data. Each of the fastq files was aligned to the hg19 build of the human genome with Cufflinks [61,62], and quality-control and result plots were generated from the Cummerbund package as described before [18]. Quality control steps investigating variability between replicates were conducted, and there were no indications of outliers (i.e., negligible variability between replicates observed).

### 3.6. Dysregulated LncRNA Characterization

The protein and non-protein coding genes whose transcripts were indicated to have been differentially expressed, at a significance level of *p* ≤ 0.01, were arranged in Venn diagrams to explore shared and unique genes among our treatment conditions. The lncRNAs with differentially expressed transcripts, at a significance level of *p* ≤ 0.01, were extracted from the total transcriptome data set and further classified using the Genomic Regions Enrichment of Annotations Tool (GREAT) to map lncRNAs whose transcripts were up- and downregulated to potential target genes based on proximity to a transcription start site (TSS) and gene annotations of the neighboring proteins [37]. The GREAT algorithm assumes that lncRNA transcription sites within 1000 kilobases (kb) of a “neighboring” gene TSS can affect the transcription of that neighboring gene. If the nearest TSS is over 1000 kb away, then no neighboring protein-coding genes are assigned. Neighboring or associated protein-coding genes are defined by the GREAT algorithm as those within 1000 kb of an input lncRNA transcription site. GREAT calculates the distance between input sequences (lncRNA genes in this case) and target TSSs by measuring the distance in nucleotide base pairs from the middle of each input sequence to the closest TSS of a protein-coding gene. GREAT was run with the binomial and hypergeometric functions disabled, but all other filters intact, since much of the input data (i.e., lncRNAs with up- and downregulated transcripts) were largely unannotated. Dysregulated lncRNAs and their associated protein-coding genes, as refined in the GREAT algorithm, were analyzed for potential functions using the Protein ANalysis THrough Evolutionary Relationships (PANTHER version 11) classification system. PANTHER uses Gene Ontology (GO)-slim terms to classify genes based on annotations established by the Gene Ontology Consortium. GO-slim is useful with large data sets, and is a viable choice when a more broad classification of gene products is desired. Gene symbols (official HUGO gene nomenclature committee (HGNC) gene symbols [35]) from our lncRNAs and neighboring protein-coding genes were also fed into the PANTHER “gene list analysis” tool to visualize and further annotate GO associations and conserved signaling pathways [40].

The web application, Idiographica version 2.3 (http://www.ncrna.org/idiographica), was used to develop chromosome maps of genes dysregulated by selected treatments and Venny version 2.0 (http://bioinfogp.cnb.csic.es/tools/venny) was used to generate Venn diagrams [36,63]. We also used the Biological Database Network’s “Database to Database Conversions” tool (https://biodbnet-abcc.ncifcrf.gov/db/db2db.php) to convert between various identifiers necessary to run specific algorithms [64] and as inputs in Microsoft Excel, Word, and Powerpoint (2013 version).

## 4. Conclusions

As seen in Supplementary Tables S6–S10, there were many other biological processes and pathways that were not discussed that are also very important in normal cellular function. Transcriptomic analyses revealed to us possible lncRNA-coding gene partnerships and their putative functions, but there is more work remaining to establish a cause and effect relationship. However, the research reported provides potential risks. Our analysis is the first of its kind to link lncRNAs to neighboring protein-coding genes (and other lncRNAs) and functions related to DEET and fipronil exposure, either alone or in combination, in primary human hepatocytes. It can help us to begin to understand the complex molecular interactions that are responsible for the human liver’s response to two common environmental chemicals at the epigenetic level. While this study was performed using primary human liver cells, we acknowledge that our findings could be different for different cell types or in different organs and tissues. Also, there are questions regarding actual tissue concentrations of insect repellents in humans, as Roy et al. [65] and others have suggested, that have yet to be resolved.

Although many of the lncRNAs we found were uncharacterized, we inferred function from factors such as chromosomal position in relation to protein-coding and noncoding genes that had been previously assigned function and associated with key molecular pathways. We also determined the relative impact of DEET and fipronil on human hepatocytes based on lncRNA expression profiles associated with each. All of this may be useful for future studies that aim to use lncRNAs in measurement of exposure to environmental chemicals as well as prognostic and diagnostic indicators of overexposure and disease. In addition, specific lncRNAs could be utilized for prevention of disease or treatments related to these and other chemicals.

This type of information is becoming more essential, as millions of new chemical compounds are synthesized each year and released into the environment at a wide range of concentrations (for varying durations) as repellents and pesticides, among other uses. We do not know what the response of another chemical or chemical combination would look like, but we felt that DEET and fipronil were a good place to start since they have different modes of action and are commonly used. The rise of the Zika epidemic and associated microcephaly in children, along with other debilitating birth defects, has prompted governmental agencies such the Centers for Disease Control (CDC) to recommend repellents that are effective at preventing disease transmission. Expecting mothers are encouraged to use DEET on their skin at a minimum concentration of 30% active ingredient every time they are at risk of being bitten by mosquitoes before and during pregnancy [4]. However, we do not really know the potential long term effects of repeated and prolonged use of these chemicals. In addition, fear and misinformation may encourage people to use DEET at higher concentrations over a longer duration to avoid contracting the disease. This could potentially be extremely harmful to unborn or infant children, the elderly, or immuno-compromised individuals along with normal healthy individuals. Finally, even though we tested specific concentrations of two common environmental chemicals, we have no data on the effects of repetitive or long-term exposure to DEET (and fipronil) on human health.

## Figures and Tables

**Table 1:** Long non-protein coding RNAs (lncRNAs) whose transcripts were significantly (*p* ≤ 0.01) differentially expressed (Diff. Exp.; up (+) or down (−) regulated) in primary human hepatocytes after exposure to 100 µM DEET, 10 µM fipronil, or a mixture of 100 µM DEET and 10 µM fipronil for 72 h.

Differential Expression	Gene Symbol ^a^	Gene Name ^b^	Log2FC (DT)	Log2FC (Fip)	Log2FC (DT+Fip)
Up	*CYP2B7P*	Cytochrome P450 family 2 subfamily B member 7, pseudogene	+2.96	+2.42	+2.79
	*HCP5*	HLA complex P5	+0.78	+0.93	+1.38
Down	*MALAT1*	Metastasis associated lung adenocarcinoma transcript 1	−1.34	−1.87	−2.28
	*NEAT1*	Nuclear paraspeckle assembly transcript 1	−1.18	−1.22	−1.52
	*LINC01554*	Long intergenic non-protein coding RNA 1554	−1.1	−1.37	−1.33
	*LINC01004*	Long intergenic non-protein coding RNA 1004	−1.58	−2.01	−2.26
	*PSORS1C3*	Psoriasis susceptibility 1 candidate 3	−1.58	−0.85	−1.39
	*AQP7P1*	Aquaporin 7 pseudogene 1	−0.8	−1.05	−0.55
	*SCART1*	Scavenger receptor protein family member	−1.49	−1.24	−1.89
	*PDXDC2P*	Pyridoxal dependent decarboxylase domain containing 2	−0.78	−1.14	−1.41
	*LINC00893*	Long intergenic non-protein coding RNA 893	−1.21	−1.18	−1.51
	*WASH5P*	WAS protein family homolog 5 pseudogene	−0.79	−0.76	−1.06
	*PFN1P2*	Profilin 1 pseudogene 2	−1.24	−1.33	−1.78
	*LINC00482*	Long intergenic non-protein coding RNA 482	−1.01	−0.91	−1.06
	*ERVK13-1*	Endogenous retrovirus group K13 member 1	−0.84	−0.78	−1.25
	*LOC100289230*	Uncharacterized LOC100289230	−1.66	−1.8	−1.44
	*LOC728040*	HCG1813624	−1.37	−2.5	−3.47
	*LOC100190986*	Uncharacterized LOC100190986	−0.91	−0.96	−1.29
	*LINC01000*	Long intergenic non-protein coding RNA 1000	−0.71	−0.53	−0.88
	*LOC100272217*	Uncharacterized LOC100272217	−1.46	−1.29	−1.54

^a^ HUGO gene nomenclature committee (HGNC) gene symbol [35]; ^b^ National Center for Biotechnology Information (NCBI) gene description; ^c^ FC = log2 fold change; DT = 100 µM DEET; Fip = 10 µM fipronil; DT + Fip = 100 µM DEET plus 10 µM fipronil mixture.

**Table 2:** Protein-coding and non-protein coding genes neighboring (within 1000 kb) the 20 lncRNAs whose transcripts were up- or downregulated by 100 µM DEET using GREAT algorithm parameters. The GREAT algorithm defines neighboring genes as those whose transcription start site (TSS) is within 1000 kb of the input lncRNAs. lncRNA = long non-protein coding RNA; kb = kilobases. All gene names are HUGO gene nomenclature committee (HGNC) gene symbols.

lncRNA	Gene(s) within 1000 kb of lncRNA
*CYP2B7P*	*CYP2A7* (−54710), *CYP2B6* (−53837), CYP2A6 *, CYP2A13 *
*HCP5*	*MICB* (−33621), *MICA* (+60915)
*AQP7P1*	*ANKRD20A1* (−646908)
*MALAT1*	*SCYL1* (−22962), *FRMD8* (+115516), NEAT1 *
*SCART1*	*CYP2E1* (−59218), *MTG1* (+67017)
*PFN1P2*	*PPIAL4B* (−247525), *NBPF9* (−199977)
*PDXDC2P*	*PDPR* (−92503), *CLEC18A* (+69943), NQO1 *
*LINC01000*	*CALU* (−88173), *METTL2B* (+174390)
*LOC100190986*	*METTL9* (−166237), *NPIPB3* (−13482)
*PSORS1C3*	*POU5F1* (−5124), *HLA-C* (+96269), HCP5 *
*LINC01554*	*GLRX* (−33468), *ELL2* (+105889)
*LOC100272217*	*FUBP3* (−1184)
*LINC00893*	*IDS* (−28345), *CXorf40A* (−6965)
*NEAT1*	*SCYL1* (−100412), *FRMD8* (+38066), MALAT1 *
*WASH5P*	*OR4F17* (−41513)
*LOC100289230*	*CHD1* (−3535)
*LINC00482*	*SLC38A10* (−10731), *TMEM105* (+24638)
*LINC01004*	*KMT2E* (−27723), *LHFPL3* (+657799)
*ERVK13-1*	*KCTD5* (−16561), *PDPK1* (+127950), RPS2 *
*LOC728040*	*AFM* (+36985), *RASSF6* (+101963), CXCL8 *

* Neighboring differentially expressed protein-coding gene or lncRNA found previously to be within 1000 kb of dysregulated lncRNA (after 100 µM DEET treatment) before the GREAT algorithm parameters were implemented.

**Table 3:** Subset of well-studied lncRNAs whose transcripts were significantly (*p* ≤ 0.01) differentially expressed (Diff. Exp.; up (+) or down (−) regulated) in primary human hepatocytes after exposure to 100 µM DEET, 10 µM fipronil, or a mixture of 100 µM DEET and 10 µM fipronil for 72 h.

Diff. Exp.	Gene Symbol ^a^	Gene Name ^b^	FC ^c^ (DT)	FC ^c^ (Fip)	FC ^c^ (DT+Fip)
Up	*H19*	Long Intergenic Non-Protein Coding RNA 8	-	+1.55	+0.59
Down	*HULC*	Highly Up-Regulated In Liver Cancer	-	−0.57	-
	*MALAT1*	Metastasis Associated Lung Adenocarcinoma Transcript 1	−1.34	−1.87	−2.28
	*NEAT1*	Nuclear Enriched Abundant Transcript 1	−1.10	−1.22	−1.52
	*XIST*	X (Inactive)-Specific Transcript	-	−0.60	−0.93
	*TSIX*	TSIX Transcript, XIST Antisense RNA	-	−0.59	−0.97
	*MEG3*	Maternally Expressed 3	-	−1.19	−1.70

^a^ HUGO gene nomenclature committee (HGNC) gene symbol [35]; ^b^ National Center for Biotechnology Information (NCBI) gene description; ^c^ FC = log2 fold change; DT = 100 µM DEET; Fip = 10 µM fipronil; DT + Fip = 100 µM DEET plus 10 µM fipronil mixture.

## Supplementary Materials

Supplementary materials can be found at www.mdpi.com/1422-0067/18/10/2104/s1.

## Abbreviations

DEET	*N*,*N*-diethyl-*m*-toluamide
NCBI	National Center for Biotechnology Information
TSS	Transcription start site
HUGO	Human Genome Organisation
HGNC	HUGO Gene Nomenclature Committee
GREAT	Genomic regions enrichment of annotations tool
PANTHER	Protein analysis through evolutionary relationships
