# Supplementary material for: Differential Expression Profile of lncRNAs from Primary Human Hepatocytes Following DEET and Fipronil Exposure
Source: Int J Mol Sci. 2017 Oct 7;18(10):2104. doi: 10.3390/ijms18102104 (PMC5666786; doi:10.3390/ijms18102104)
Supplement: Supplementary file 1 [file ijms-18-02104-s001.pdf]

SUPPLEMENTARY MATERIAL

Differential Expression Profile of lncRNAs from Primary Human Hepatocytes Following DEET and Fipronil Exposure

FIGURES

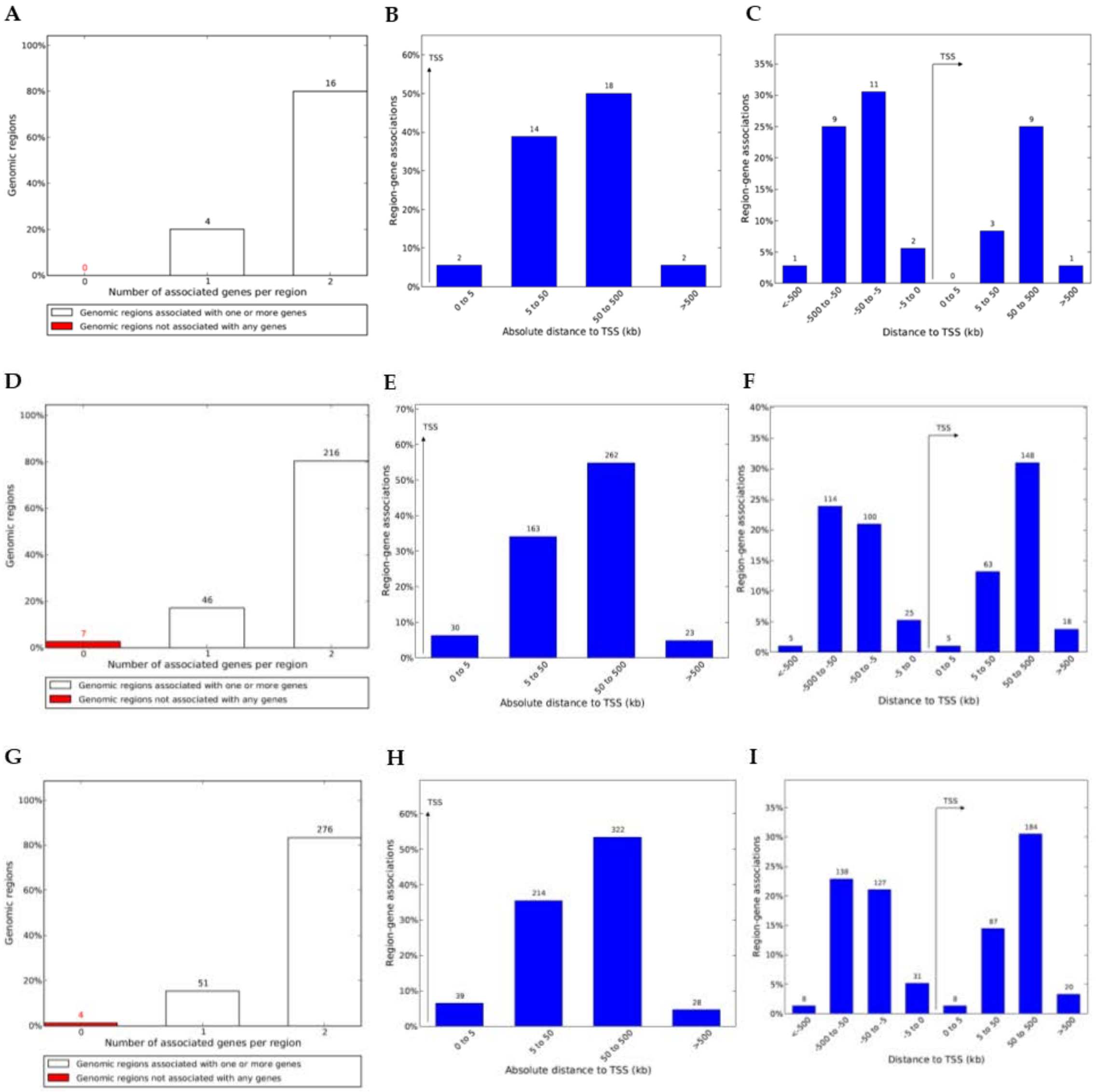

Figure S1. Genomic relationship between lncRNA transcription sites whose transcripts were differentially expressed in primary human hepatocytes after exposure to 100 μM DEET to the nearest protein-coding gene transcription start site (TSS) that lies within 1000 kilobases (kb) of the lncRNA. If the nearest TSS was over 1000 kb then no neighboring protein-coding genes were assigned. (A) Number of protein-coding genes associated with up- or downregulated lncRNA transcription sites (referred to as genomic regions in the graphs) after 100 μM DEET exposure; values in (A) that are red indicate the number of genomic regions (i.e., lncRNAs) that do not lie within 1000 kb of a protein-coding gene TSS. Percentages on the Y axis in (A) refer to the ratio of differentially expressed lncRNAs that neighbor 0, 1, or 2 or more protein-coding genes to the total number of lncRNAs dysregulated by 100 μM DEET (20 total in this case). (B) Genomic distance in kb

of lncRNA transcription sites with up- or down-regulated transcripts after 100 μM DEET exposure to closest protein-coding gene TSS; percentages on the Y axis in (B) refer to the ratio of lncRNAs that fall into categories within a certain range from the TSS of the closest protein-coding gene to the total number of lncRNAs that fall within these ranges (36 total since a single gene can span more than one range category). (C) Genomic distance in kb and orientation of lncRNA transcription sites with up- or down-regulated transcripts after 100 μM DEET exposure to closest protein-coding gene TSS. Percentages on the Y axis in (C) refer to the ratio of lncRNAs that fall into categories within a certain range both before and after the closest protein-coding gene TSS to the total number of lncRNAs that fall within these ranges (36 total in this case). D-F represents the same data points for the 10 μM fipronil treatment and G-I represents the same data points for the 100 μM DEET plus 10 μM fipronil treatment.

TABLES

Table S1. Chromosome (chrom) distribution of lncRNAs significantly dysregulated ($P \leq 0.01$) after primary human hepatocytes were exposed to 100 μM DEET (DT), 10 μM fipronil (Fip), or a mixture of 100 μM DEET and 10 μM fipronil (DT+Fip).

Chrom	Total[a] Genes	lncRNA per chrom (DT)	lncRNA per chrom (Fip)	lncRNA per chrom (DT+Fip)	lncRNA vs totalb (DT)	lncRNA vs totalb (Fip)	lncRNA vs totalb (DT+Fip)
**1**	5166	1	27	35	0.02%	0.52%	0.68%
**2**	3920	0	15	17	0.00%	0.38%	0.43%
**3**	2984	0	13	16	0.00%	0.44%	0.54%
**4**	2468	1	11	15	0.04%	0.45%	0.61%
**5**	2795	2	14	18	0.07%	0.50%	0.64%
**6**	2827	2	16	23	0.07%	0.57%	0.81%
**7**	2830	2	22	29	0.07%	0.78%	1.02%
**8**	2321	0	7	7	0.00%	0.30%	0.30%
**9**	2224	2	15	15	0.09%	0.67%	0.67%
**10**	2173	1	17	19	0.05%	0.78%	0.87%
**11**	3159	2	12	13	0.06%	0.38%	0.41%
**12**	2841	0	14	12	0.00%	0.49%	0.42%
**13**	1275	0	2	3	0.00%	0.16%	0.24%
**14**	2204	0	5	8	0.00%	0.23%	0.36%
**15**	2105	0	12	15	0.00%	0.57%	0.71%
**16**	2375	3	16	21	0.13%	0.67%	0.88%
**17**	2896	1	8	17	0.03%	0.28%	0.59%
**18**	1120	0	4	4	0.00%	0.36%	0.36%
**19**	2852	2	14	16	0.07%	0.49%	0.56%
**20**	1376	0	5	6	0.00%	0.36%	0.44%
**21**	819	0	5	4	0.00%	0.61%	0.49%
**22**	1309	0	5	10	0.00%	0.38%	0.76%
**X**	2345	1	8	8	0.04%	0.34%	0.34%
	**56384**	**20**	**267**[c]	**331**[c]			

[a] Total coding and noncoding genes based on Ensembl release 87 [25].
[b] LncRNAs vs total = the number of dysregulated lncRNAs from a single chromosome divided by the total number of known genes, coding and noncoding, on that chromosome (represented as a percentage).
[c] Two genes omitted since chromosome location not well-established.

Table S2. Chromosomal distribution of protein-coding genes significantly dysregulated ($P \leq 0.01$) after primary human hepatocytes were exposed to 100 μM DEET (DT), 10 μM fipronil (Fip), or a mixture of 100 μM DEET and 10 μM fipronil (DT+Fip).

Chrom	Genes Total[a]	Coding Genes (DT)	Coding genes (Fip)	Coding genes (DT+Fip)	Coding vs total[b] (DEET)	Coding vs total[b] (Fip)	Coding vs total[b] (DT+Fip)
**1**	5166	26	409	568	0.50%	7.92%	10.99%
**2**	3920	5	222	328	0.13%	5.66%	8.37%
**3**	2984	11	198	264	0.37%	6.64%	8.85%
**4**	2468	11	158	209	0.45%	6.40%	8.47%
**5**	2795	8	156	224	0.29%	5.58%	8.01%
**6**	2827	9	202	277	0.32%	7.15%	9.80%
**7**	2830	16	193	264	0.57%	6.82%	9.33%
**8**	2321	6	139	186	0.26%	5.99%	8.01%
**9**	2224	9	150	207	0.40%	6.74%	9.31%
**10**	2173	9	151	175	0.41%	6.95%	8.05%
**11**	3159	7	208	298	0.22%	6.58%	9.43%
**12**	2841	4	214	290	0.14%	7.53%	10.21%
**13**	1275	1	49	71	0.08%	3.84%	5.57%
**14**	2204	1	104	156	0.05%	4.72%	7.08%
**15**	2105	8	114	160	0.38%	5.42%	7.60%
**16**	2375	10	178	242	0.42%	7.49%	10.19%
**17**	2896	5	183	291	0.17%	6.32%	10.05%
**18**	1120	2	53	68	0.18%	4.73%	6.07%
**19**	2852	12	256	350	0.42%	8.98%	12.27%
**20**	1376	4	81	128	0.29%	5.89%	9.30%
**21**	819	1	37	48	0.12%	4.52%	5.86%
**22**	1309	4	90	132	0.31%	6.88%	10.08%
**X**	2345	3	125	164	0.13%	5.33%	6.99%
	**56384**	**172**	**3670**[c]	**5100**[c]			

[a] Total coding and noncoding genes based on Ensembl release 87 [25].

[b] Coding vs total = the number of dysregulated coding genes from a single chromosome divided by the total number of known genes, coding and noncoding, on that chromosome (represented as a percentage).

[c] Two genes omitted since chromosome location not well-established

.

Table S3. LncRNAs that were differentially expressed after primary human hepatocytes were treated with 100 µM DEET.

Differentially Expressed lncRNAs (100 µM DEET only)

Gene symbol	Genbank Accession	Chromosome Coordinates	Gene Expression	LncRNA Associated Genes
CYP2B7P	NR_001278	chr19 : 41430169 - 41456565	Up	CYP2A7 (-54710), CYP2B6 (-53837), CYP2A6*, CYP2A13*
HCP5	NR_040662	chr6 : 31430956 - 31433586	Up	MICB (-33621), MICA (+60915)
AQP7P1	NR_002817	chr9 : 67270214 - 67289492	Down	ANKRD20A1 (-646908)
PFN1P2	NR_003242	chr1 : 144610814 - 144612727	Down	PPIAL4B (-247525), NBPF9 (-199977)
PDXDC2P	NR_003610	chr16 : 70010201 - 70099851	Down	PDPR (-92503), CLEC18A (+69943), NQO1*
WASH5P	NR_033266	chr19 : 60950 - 70966	Down	OR4F17 (-41513)
SCART1	NR_002934	chr10 : 135267431 - 135281953	Down	CYP2E1 (-59218), MTG1 (+67017)
MALAT1	NR_002819	chr11 : 65265232 - 65273939	Down	SCYL1 (-22962), FRMD8 (+115516), NEAT1*
LINC01000	NR_024368	chr7 : 128281294 - 128301052	Down	CALU (-88173), METTL2B (+174390)
LOC100190986	NR_024456	chr16 : 21443344 - 21445776	Down	METTL9 (-166237), NPIPB3 (-13482)
PSORS1C3	NR_026816	chr6 : 31141511 - 31145676	Down	POU5F1 (-5124), HLA-C (+96269), HCP5*
LINC01554	NR_026936	chr5 : 95187935 - 95195836	Down	GLRX (-33468), ELL2 (+105889)
LOC100272217	NR_027440	chr9 : 133452736 - 133454881	Down	FUBP3 (-1184)
LINC00893	NR_027455	chrX : 148609131 - 148621312	Down	IDS (-28345), CXorf40A (-6965)
NEAT1	NR_028272	chr11 : 65190268 - 65194003	Down	SCYL1 (-100412), FRMD8 (+38066), MALAT1*
LOC100289230	NR_036530	chr5 : 98264837 - 98266713	Down	CHD1 (-3535)
LINC00482	NR_038080	chr17 : 79276623 - 79283048	Down	SLC38A10 (-10731), TMEM105 (+24638)
LINC01004	NR_039981	chr7 : 104622193 - 104631612	Down	KMT2E (-27723), LHFPL3 (+657799)
ERVK13-1	NR_040023	chr16 : 2708389 - 2723440	Down	KCTD5 (-16561), PDPK1 (+127950), RPS2*
LOC728040	NR_046377	chr4 : 74374519 - 74394250	Down	AFM (+36985), RASSF6 (+101963), CXCL8*

Plus (+) means upstream while minus (-) means downstream.

Table S4. LncRNAs that were differentially expressed after primary human hepatocytes were treated with 10 µM Fipronil.

Differentially Expressed lncRNAs (10 µM Fipronil only)

Gene symbol	Genbank Accession	Chromosome Coordinates	Gene Expression	LncRNA Associated Genes
RPL23AP7	NR_000029	chr2 : 114368815 - 114384715	Up	RABL2A (-8041), FOXD4L1 (+120104)
CYP2B7P	NR_001278	chr19 : 41430169 - 41456565	Up	CYP2A7 (-54710), CYP2B6 (-53837)
MT1L	NR_001447	chr16 : 56651372 - 56652730	Up	MT1E (-7336), MT2A (+9940)
MGC72080	NR_002822	chr7 : 97595907 - 97601638	Up	ASNS (-96919), OCM2 (+20735)
SNX29P2	NR_002939	chr16 : 29313607 - 29376380	Up	NPIPB11 (+70356), LAT (+348847)
HSD17B7P2	NR_003086	chr10 : 38645307 - 38667433	Up	ZNF37A (+273106)
GOLGA6L5P	NR_003246	chr15 : 85045805 - 85060078	Up	ZSCAN2 (-91294), ADAMTSL3 (+730104)
RRN3P1	NR_003370	chr16 : 21807950 - 21830495	Up	NPIPB4 (+49516), OTOA (+129388)
RP9P	NR_003500	chr7 : 32956426 - 32982782	Up	FKBP9 (-27413), AVL9 (+434503)
GTF2IP4	NR_003580	chr7 : 72569011 - 72621336	Up	STAG3L3 (-118930), NSUN5 (+127639)
LOC728554	NR_003615	chr5 : 177302261 - 177311269	Up	PROP1 (+116478), B4GALT7 (+279664)
MT1DP	NR_003658	chr16 : 56677598 - 56678853	Up	MT1B (-7585), MT1A (+5648)
WASH3P	NR_003659	chr15 : 102501015 - 102519296	Up	OR4F4 (-46858)
RPSAP58	NR_003662	chr19 : 23945815 - 24010919	Up	ZNF726 (-119325), RPSAP58 (+32551)
FGF7P6	NR_003674	chr9 : 46687556 - 46746820	Up	NONE
PI4KAP2	NR_003700	chr22 : 21827286 - 21871780	Up	UBE2L3 (-54203), HIC2 (+52784)
FKBP9P1	NR_003949	chr7 : 55748766 - 55772260	Up	VOPP1 (-120295), SEPT14 (+169969)
LOC154761	NR_015421	chr7 : 143509060 - 143533810	Up	OR2F2 (-110824), CTAGE6 (-66646)
CYP2D7	NR_002570	chr22 : 42536213 - 42540575	Up	CYP2D6 (-11486), TCF20 (+73054)
GUCY2EP	NR_024042	chr11 : 76391209 - 76432833	Up	TSKU (-90775), LRRC32 (-30977)
WASH2P	NR_024077	chr2 : 114341229 - 114361294	Up	RABL2A (-33544), FOXD4L1 (+94601)
RASA4CP	NR_024116	chr7 : 44068485 - 44083895	Up	DBNL (-8120), UBE2D4 (+110147)
MSL3P1	NR_024322	chr2 : 234774089 - 234777055	Up	TRPM8 (-50471), HJURP (-12360)
FBLL1	NR_024356	chr5 : 167956581 - 167957639	Up	FBLL1 (+989)
GOLGA2P7	NR_027001	chr15 : 84860599 - 84898920	Up	ZSCAN2 (-264476), ADAMTSL3 (+556922)
LOC654342	NR_027238	chr2 : 91824708 - 91847975	Up	NONE
SERPINB9P1	NR_033851	chr6 : 2854890 - 2876744	Up	SERPINB1 (-23577), SERPINB9 (+37697)

LOC389765	NR_029410	chr9 : 88420916 - 88457794	Up	NAA35 (-116706), AGTPBP1 (-82622)
ROCK1P1	NR_033770	chr18 : 109064 - 122222	Up	TUBB8P12 (-66086), USP14 (-42965)
TEKT4P2	NR_038327	chr21 : 9907188 - 9968593	Up	NONE
WHAMMP1	NR_036650	chr15 : 32812048 - 32825942	Up	GOLGA8O (-71160), GOLGA8N (-66662)
MAFIP	NR_046439	chr4 : 53588 - 115071	Up	ZNF595 (+31120), ZNF732 (+214780)
STAG3L2	NR_040584	chr7 : 74298262 - 74306731	Up	GTF2IRD2 (-34650), WBSCR16 (+187132)
LOC100288778	NR_130745	chr12 : 87983 - 91263	Up	IQSEC3 (-86308)
GAS5	NR_002578	chr1 : 173832385 - 173837125	Up	ZBTB37 (-2738)
SNHG6	NR_002599	chr8 : 67782983 - 67837777	Up	MCMDC2 (+27396), TCF24 (+64445)
H19	NR_002196	chr11 : 2016405 - 2019065	Up	MRPL23 (+49227), IGF2 (+144733)
SNHG5	NR_003038	chr6 : 86386724 - 86388451	Up	SYNCRIP (-34585)
EPB41L4A-AS1	NR_015370	chr5 : 111496222 - 111498198	Up	NREP (-184582), EPB41L4A (+257803)
UCA1	NR_015379	chr19 : 15939756 - 15947131	Up	OR10H1 (-24508), CYP4F2 (+65486)
MIR4435-2HG	NR_015395	chr2 : 112124590 - 112252692	Up	BCL2L11 (+310135), ANAPC1 (+453626)
ZMIZ1-AS1	NR_015429	chr10 : 80703082 - 80827205	Up	ZMIZ1 (-63648), RPS24 (+971626)
UBA6-AS1	NR_015439	chr4 : 68566995 - 68588222	Up	UBA6 (-10712), GNRHR (+42469)
DANCR	NR_024031	chr4 : 53578620 - 53580305	Up	USP46 (-53961), ERVMER34-1 (+38344)
DLGAP1-AS1	NR_024101	chr18 : 3496029 - 4455266	Up	DLGAP1 (+479687), TGIF1 (+523973)
CYTOR	NR_024204	chr2 : 87754973 - 87821030	Up	PLGLB1 (-539027), PLGLB2 (-259604)
LINC01512	NR_024478	chr6 : 43858764 - 43905944	Up	TMEM63B (-212297), VEGFA (+143910)
LOC100268168	NR_026682	chr5 : 172381785 - 172386371	Up	RPL26L1 (-1818)
LINC00239	NR_026774	chr14 : 102196773 - 102198862	Up	PPP2R5C (-30317), DIO3 (+170130)
URB1-AS1	NR_026845	chr21 : 33765441 - 33766266	Up	URB1 (-519)
LOC100133669	NR_026913	chr8 : 144063447 - 144099807	Up	CYP11B2 (-82368), LY6E (-18318)
LOC115110	NR_026927	chr1 : 2481358 - 2484284	Up	TNFRSF14 (-4984)
DRAIC	NR_026979	chr15 : 69854058 - 69863779	Up	RPLP1 (+113796), TLE3 (+531596)
LOC153910	NR_027311	chr6 : 142847591 - 142959026	Up	GPR126 (+280243), HIVEP2 (+363029)
LINC00938	NR_028408	chr12 : 46119502 - 46121704	Up	ARID2 (-2845)
LOC642361	NR_029407	chr10 : 81585657 - 81587358	Up	SFTPD (+122351), SFTPA1 (+215807)
LOC284344	NR_033888	chr19 : 43715942 - 43752798	Up	PSG4 (-24444), PSG9 (+39310)
LOC729970	NR_033998	chr1 : 95393583 - 95428826	Up	CNN3 (-18371), ALG14 (+127296)

LINC00673	NR_036488	chr17 : 70399462 - 70588943	Up	SOX9 (+377042), SLC39A11 (+594624)
LOC100294145	NR_037177	chr6 : 32861952 - 32871535	Up	HLA-DMB (+42103), PSMB9 (+44806)
MIR210HG	NR_038262	chr11 : 565656 - 568457	Up	PHRF1 (-9464), RASSF7 (+6005)
LOC100506844	NR_038269	chr12 : 58325231 - 58329947	Up	CTDSP2 (-87067), XRCC6BP1 (-7771)
LINC00888	NR_038301	chr3 : 183165395 - 183173800	Up	MCF2L2 (-23535), KLHL6 (+103879)
OSER1-AS1	NR_038337	chr20 : 42839599 - 42854667	Up	JPH2 (-30915), FITM2 (+92676)
LOC100507389	NR_038455	chr3 : 142645516 - 142661378	Up	PCOLCE2 (-45402), PAQR9 (+28731)
LYPLAL1-AS1	NR_038845	chr1 : 219254316 - 219347130	Up	LYPLAL1 (-46463), TGFB2 (+781146)
IL10RB-AS1	NR_038974	chr21 : 34637936 - 34638565	Up	IL10RB (-412)
LINC00862	NR_040064	chr1 : 200311671 - 200342920	Up	ZNF281 (+51888), NR5A2 (+330566)
LINC00266-1	NR_040415	chr20 : 62921737 - 62934707	Up	PCMTD2 (+41101)
HCP5	NR_040662	chr6 : 31430956 - 31433586	Up	MICB (-33621), MICA (+60915)
HEIH	NR_045680	chr5 : 180256953 - 180262726	Up	MGAT1 (-23011), ZFP62 (+28437)
SH3BP5-AS1	NR_046084	chr3 : 15295690 - 15382901	Up	SH3BP5 (+34840), CAPN7 (+91546)
RCHY1	NM_001008925	chr4 : 76404246 - 76439640	Up	THAP6 (-17740), PARM1 (+563617)
LINC01587	NM_005750	chr4 : 5526882 - 5529527	Up	C4orf6 (+1322), EVC2 (+182089)
LINC01619	NM_001256373	chr12 : 92378751 - 92539673	Up	DCN (-882312), BTG1 (+80461)
PCAT6	NR_046325	chr1 : 202780073 - 202781041	Up	KDM5B (-1959)
HLA-DRB6	NR_001298	chr6 : 32520489 - 32527779	Down	HLA-DRB5 (-26070), HLA-DRB1 (+33491)
HLA-H	NR_001434	chr6 : 29855382 - 29858856	Down	HLA-A (-51918), HLA-G (+62363)
ANKRD20A12P	NR_046228	chr1 : 142697420 - 142713605	Down	NONE
TPTEP1	NR_001591	chr22 : 17082800 - 17129720	Down	CCT8L2 (-32560), XKR3 (+196329)
CMAHP	NR_002174	chr6 : 25081294 - 25138620	Down	FAM65B (-198762), LRRC16A (-169699)
NPY6R	NR_002713	chr5 : 137136881 - 137146439	Down	MYOT (-61820), HNRNPA0 (-51621)
MBL1P	NR_002724	chr10 : 81664653 - 81691557	Down	SFTPD (+30754), SFTPA1 (+307404)
AQP7P1	NR_002817	chr9 : 67270214 - 67289492	Down	ANKRD20A1 (-646908)
AKR7L	NR_040288	chr1 : 19592475 - 19600568	Down	EMC1 (-18476), AKR7A3 (+19222)
HERC2P4	NR_109773.1	chr16 : 32162608 - 32163874	Down	ZNF267 (+278162), TP53TG3 (+524812)
PGM5P2	NR_002836	chr9 : 69080243 - 69147854	Down	FOXD4L6 (+88155)
SCART1	NR_002934	chr10 : 135267431 - 135281953	Down	CYP2E1 (-59218), MTG1 (+67017)
RPL32P3	NR_003111	chr3 : 129101676 - 129118282	Down	H1FX (-74859), EFCAB12 (+37515)

RNF5P1	NR_003129	chr8 : 38457692 - 38458775	Down	TACC1 (-186488), FGFR1 (-132871)
PFN1P2	NR_003242	chr1 : 144610814 - 144612727	Down	PPIAL4B (-247525), NBPF9 (-199977)
LOC220729	NR_003266	chr3 : 197340897 - 197354752	Down	BDH1 (-64967), KIAA0226 (+115765)
CES1P1	NR_003276	chr16 : 55794510 - 55808826	Down	CES1 (+65402), SLC6A2 (+111112)
EP400NL	NR_003290	chr12 : 132568827 - 132610885	Down	DDX51 (+39024), EP400 (+155348)
GGT8P	NR_003503	chr2 : 91963367 - 91970153	Down	NONE
GUSBP2	NR_003504	chr6 : 26839265 - 26924333	Down	ZNF322 (-221819), HIST1H2BJ (+218730)
DPY19L2P2	NR_003561	chr7 : 102815461 - 102920913	Down	NAPEPLD (-78558), PMPCB (-69682)
PDXDC2P	NR_003610	chr16 : 70010201 - 70099851	Down	PDPR (-92503), CLEC18A (+69943)
UBE2Q2P1	NR_003661	chr15 : 85070426 - 85123412	Down	ZSCAN2 (-47317), ADAMTSL3 (+774081)
SPDYE7P	NR_003666	chr7 : 72333317 - 72339655	Down	CALN1 (-459128), POM121 (-13450)
PPIEL	NR_003929	chr1 : 39957317 - 40025370	Down	BMP8A (+34026), PABPC4 (+50729)
LOC652276	NR_015441	chr16 : 2653384 - 2680495	Down	KCTD5 (-65536), PDPK1 (+78975)
CROCCP3	NR_023386	chr1 : 16793930 - 16819196	Down	NECAP2 (+39332), NBPF1 (+133419)
CLUHP3	NR_024034	chr16 : 31711933 - 31718745	Down	ZNF720 (-9216), AHSP (+176154)
HLA-J	NR_024240	chr6 : 29968787 - 30028961	Down	ZNRD1 (-30157), HLA-A (+89837)
LOC155060	NR_036573	chr7 : 148982371 - 148994403	Down	ZNF783 (+29125), ZNF777 (+169827)
ADAM1A	NR_036636	chr12 : 112336866 - 112339706	Down	MAPKAPK5 (+57856), TMEM116 (+112629)
MT1JP	NR_036677	chr16 : 56669650 - 56670998	Down	MT1A (-2254)
AZGP1P1	NR_036679	chr7 : 99578384 - 99581860	Down	ZKSCAN1 (-33082), AZGP1 (-6342)
LOC730102	NR_037167	chr1 : 177975274 - 178007142	Down	RASAL2 (-72068), SEC16B (-52158)
ESPNP	NR_026567	chr1 : 17017712 - 17046652	Down	CROCC (-216263), NBPF1 (-92200)
FAM86B3P	NR_024361	chr8 : 8086091 - 8102387	Down	SGK223 (+149769), ZNF705B (+293095)
LOC728989	NR_024442	chr1 : 146490894 - 146514599	Down	NBPF12 (+128691), PRKAB2 (+141376)
BMS1P4	NR_026592	chr10 : 75458908 - 75490272	Down	SEC24C (-29559), AGAP5 (-17036)
HTATSF1P2	NR_033884	chr6 : 3020389 - 3025005	Down	RIPK1 (-45961), NQO2 (+22489)
GOLGA2P5	NR_024261	chr12 : 100550174 - 100567121	Down	ANKS1B (-180633), ACTR6 (-35217)
ZNF37BP	NR_026777	chr10 : 43008960 - 43048280	Down	ZNF33B (+105372)
ANKRD36BP1	NR_026844	chr1 : 168214818 - 168216668	Down	TBX19 (-34535), SFT2D2 (+20497)
FAM45BP	NR_027141	chrX : 129628914 - 129631421	Down	RBMX2 (+94225), ENOX2 (+407040)
GUSBP3	NR_027386	chr5 : 68935289 - 69006272	Down	SERF1B (-350315), GTF2H2C (+114712)

MTMR9LP	NR_026850	chr1 : 32697260 - 32707311	Down	LCK (-14554), EIF3I (+14315)
FRG1JP	NR_033907	chr9 : 68427782 - 68454375	Down	ANKRD20A1 (+514318), FOXD4L6 (+761125)
BEND3P3	NR_027512	chr10 : 81442730 - 81448650	Down	SFTPA1 (+74989), SFTPD (+263169)
ARHGAP27P1-BPTFP1-KPNA2P3	NR_026899	chr17 : 62745779 - 62778117	Down	SMURF2 (-103762), LRRC37A3 (+152955)
AKR1C8P	NR_027916	chr10 : 5196654 - 5227150	Down	AKR1CL1 (+15194), AKR1C3 (+75917)
RPL23AP87	NR_029406	chr17 : 81174665 - 81188573	Down	METRNL (+144052)
ANKRD20A9P	NR_027995	chr13 : 19408542 - 19446109	Down	TUBA3C (+328666)
PMS2P9	NR_028058	chr7 : 76668796 - 76682355	Down	POMZP3 (-419018), FGL2 (+153567)
GLUD1P3	NR_048575	chr10 : 75491298 - 75495367	Down	AGAP5 (-35779), SEC24C (-10816)
GCSHP3	NR_033248	chr2 : 206980296 - 206981296	Down	INO80D (-29890), NDUFS1 (+43122)
WASH5P	NR_033266	chr19 : 60950 - 70966	Down	OR4F17 (-41513)
CTSLP2	NR_033407	chr10 : 48155942 - 48158691	Down	ASAH2C (-102299), AGAP9 (-58519)
GTF2H2B	NR_033417	chr5 : 69711196 - 69746189	Down	SERF1A (-467821), SMN2 (+383254)
YY1P2	NR_033658	chr2 : 139654893 - 139656744	Down	NXPH2 (-117901)
DNM1P41	NR_033787	chr15 : 85045805 - 85060078	Down	ZSCAN2 (-91294), ADAMTSL3 (+730104)
CEACAM22P	NR_027754	chr19 : 45041044 - 45060150	Down	IGSF23 (-66343), ZNF180 (-46023)
SMG1P7	NR_033959	chr16 : 70253483 - 70259936	Down	EXOSC6 (+29123), CLEC18C (+48782)
ZNF767P	NR_027788	chr7 : 149244244 - 149321881	Down	KRBA1 (-129085), ZNF746 (-88165)
HERC2P7	NR_036470	chr15 : 23390721 - 23393943	Down	GOLGA8S (-207661), GOLGA8I (+137090)
LOC100131257	NR_034022	chr7 : 7115400 - 7136417	Down	CCZ1B (-259508), C1GALT1 (-96337)
TMEM198B	NR_036476	chr12 : 56211805 - 56236767	Down	DNAJC14 (+279)
ALG1L9P	NR_073386	chr11 : 71498556 - 71524905	Down	KRTAP5-11 (-217810), DEFB108B (-32515)
PRKXP1	NR_073405	chr15 : 101087956 - 101099488	Down	ASB7 (-49017), CERS3 (-8797)
LOC100130075	NR_073494	chr12 : 69198216 - 69199274	Down	MDM2 (-3211)
LOC202181	NR_026921	chr5 : 177045500 - 177099278	Down	B4GALT7 (+45288), PROP1 (+350854)
AKR1C6P	NR_026743	chr10 : 4913858 - 4958465	Down	AKR1C1 (-69283), AKR1E2 (+67716)
MALAT1	NR_002819	chr11 : 65265232 - 65273939	Down	SCYL1 (-22962), FRMD8 (+115516)
HULC	NR_004855	chr6 : 8435855 - 8785678	Down	SLC35B3 (-175051)
XIST	NR_001564	chrX : 73012039 - 73072588	Down	ZCCHC13 (-481711), CHIC1 (+259270)

MEG3	NR_002766	chr14 : 101292444 - 101327360	Down	RTL1 (+41282), DLK1 (+116738)
TSIX	NR_003255	chrX : 73012039 - 73072588	Down	ZCCHC13 (-481711), CHIC1 (+259270)
NEAT1	NR_028272	chr11 : 65190268 - 65194003	Down	SCYL1 (-100412), FRMD8 (+38066)
LINC00115	NR_024321	chr1 : 761585 - 762902	Down	OR4F16 (-140191), SAMD11 (-98874)
HNF1A-AS1	NR_024345	chr12 : 121407640 - 121410095	Down	SPPL3 (-66694), HNF1A (-7478)
LINC01000	NR_024368	chr7 : 128281294 - 128301052	Down	CALU (-88173), METTL2B (+174390)
LINC01018	NR_024423	chr5 : 6582286 - 6588613	Down	NSUN2 (+47954), UBE2QL1 (+136714)
PTGES2-AS1	NR_024425	chr9 : 130890807 - 130892913	Down	PTGES2 (-1119)
MIR100HG	NR_024430	chr11 : 121959810 - 122073770	Down	UBASH3B (-509593), BLID (-29867)
PVT1	NR_003367	chr8 : 128902873 - 129113499	Down	TMEM75 (-47595)
LINC00926	NR_024433	chr15 : 57592562 - 57599967	Down	CGNL1 (-72438), TCF12 (+385444)
LOC100128288	NR_024447	chr17 : 8261730 - 8263859	Down	KRBA2 (+12063), ODF4 (+19613)
NDUFB2-AS1	NR_024454	chr7 : 140395135 - 140406446	Down	NDUFB2 (+4320), BRAF (+223773)
LOC100190986	NR_024456	chr16 : 21443344 - 21445776	Down	METTL9 (-166237), NPIPB3 (-13482)
RAMP2-AS1	NR_024461	chr17 : 40905946 - 40915060	Down	RAMP2 (-2709)
PWARSN	NR_022011	chr15 : 25227140 - 25228937	Down	SNURF (+27904), UBE3A (+425756)
ZNF674-AS1	NR_015378	chrX : 46404924 - 46407910	Down	ZNF674 (-1541)
LOC100128573	NR_024491	chr19 : 7537722 - 7538247	Down	PEX11G (+15920), ARHGEF18 (+33411)
FLJ42627	NR_024492	chr16 : 2688982 - 2696130	Down	KCTD5 (-39920), PDPK1 (+104591)
LINC00999	NR_024497	chr10 : 38717073 - 38741081	Down	ZNF37A (+345813)
MIR600HG	NR_026677	chr9 : 125871772 - 125877756	Down	GPR21 (+77958), STRBP (+156091)
LINC01558	NR_026773	chr6 : 168185218 - 168197539	Down	TCP10 (-393425), C6orf123 (+6160)
LINC00574	NR_026780	chr6 : 170188885 - 170202969	Down	C6orf70 (+44206), DLL1 (+403634)
PSORS1C3	NR_026816	chr6 : 31141511 - 31145676	Down	POU5F1 (-5124), HLA-C (+96269)
LINC00174	NR_026873	chr7 : 65841030 - 65865395	Down	KCTD7 (-352430), TPST1 (+183027)
LINC01554	NR_026936	chr5 : 95187935 - 95195836	Down	GLRX (-33468), ELL2 (+105889)
CDKN2B-AS1	NR_003529	chr9 : 21994789 - 22121093	Down	DMRTA1 (-388899), CDKN2B (-48579)
LINC00663	NR_026956	chr19 : 19867180 - 19887222	Down	ZNF14 (-33295), ZNF506 (+55359)
LOC143666	NR_026967	chr11 : 573807 - 575885	Down	PHRF1 (-1675)
LOC90784	NR_026984	chr2 : 86247338 - 86250991	Down	ST3GAL5 (-133028), POLR1A (+84113)
LINC00923	NR_024172	chr15 : 98285845 - 98417659	Down	ARRDC4 (-152176)

LOC100132111	NR_024237	chr1 : 151810338 - 151816641	Down	RORC (-9142), THEM5 (+12683)
LINC00265	NR_026999	chr7 : 39773166 - 39834222	Down	CDK13 (-185942), RALA (+140612)
LOC93429	NR_027003	chr19 : 46713498 - 46718094	Down	IGFL1 (-17213), IGFL2 (+64757)
THUMPD3-AS1	NR_027007	chr3 : 9430536 - 9439174	Down	THUMPD3 (+30119), LHFPL4 (+160631)
ESRG	NR_027122	chr3 : 54156692 - 55108584	Down	LRTM1 (+329463), CACNA2D3 (+475945)
LINC01126	NR_027251	chr2 : 43454349 - 43455994	Down	ZFP36L2 (-1424)
MGC27382	NR_027310	chr1 : 78695282 - 78835147	Down	PTGFR (-191542), GIPC2 (+253629)
MZF1-AS1	NR_027334	chr19 : 59070552 - 59086164	Down	UBE2M (-8031), MZF1 (+6584)
LINC00910	NR_027412	chr17 : 41447212 - 41466266	Down	ARL4D (-19588), TMEM106A (+92845)
LOC100272217	NR_027440	chr9 : 133452736 - 133454881	Down	FUBP3 (-1184)
MAN1B1-AS1	NR_027447	chr9 : 139979397 - 139981269	Down	MAN1B1 (-1046)
LINC00893	NR_027455	chrX : 148609131 - 148621312	Down	IDS (-28345), CXorf40A (-6965)
LINC00894	NR_027456	chrX : 149106765 - 149185018	Down	MAMLD1 (-385794), MAGEA8 (+135951)
TAPT1-AS1	NR_027696	chr4 : 16228285 - 16259810	Down	TAPT1 (-15884), LDB2 (+656384)
MIR99AHG	NR_027790	chr21 : 17442841 - 17982094	Down	USP25 (+610124)
LOC284412	NR_029390	chr19 : 37756840 - 37759912	Down	HKR1 (-67204), ZNF383 (+49194)
LINC01347	NR_029401	chr1 : 243219615 - 243265046	Down	PLD5 (-554511), CEP170 (+176021)
LOC643406	NR_029405	chr20 : 5451841 - 5457780	Down	PROKR2 (-157433), GPCPD1 (+136861)
TLR8-AS1	NR_030727	chrX : 12920935 - 12961419	Down	TMSB4X (-52052), TLR8 (+16438)
SLC25A25-AS1	NR_033374	chr9 : 130873449 - 130881013	Down	PTGES2-AS1 (-13381), SLC25A25 (+46751)
DKFZP434I0714	NR_033797	chr4 : 153457415 - 153460415	Down	FBXW7 (-1662)
WAC-AS1	NR_033805	chr10 : 28808845 - 28821283	Down	MPP7 (-245993), WAC (-7284)
FAM83H-AS1	NR_033849	chr8 : 144816309 - 144828507	Down	FAM83H (-6437), SCRIB (+75141)
LOC283177	NR_033852	chr11 : 134306375 - 134375555	Down	B3GAT1 (-78718)
LINC00514	NR_033861	chr16 : 3039054 - 3044510	Down	CLDN9 (-20675), PKMYT1 (-11242)
LINC01252	NR_033890	chr12 : 11700963 - 11717335	Down	PRB2 (-160650), ETV6 (-93639)
LINC00921	NR_033904	chr16 : 3313767 - 3317566	Down	ZNF263 (-17276), MEFV (-9040)
LOC100132077	NR_033937	chr9 : 97094757 - 97123230	Down	HIATL1 (-27839), ZNF169 (+87401)
LINC00842	NR_033957	chr10 : 47096453 - 47151400	Down	NPY4R (+40393), ANXA8L1 (+50113)
FLJ22763	NR_033977	chr3 : 108855560 - 108868951	Down	MORC1 (-25267), DPPA2 (+173108)
LINC01502	NR_034016	chr9 : 138466770 - 138478958	Down	PAEP (+19260), GLT6D1 (+58522)

LINC00885	NR_034088	chr3 : 195869506 - 195887761	Down	TFRC (-69574), ZDHHC19 (+59632)
CCDC18-AS1	NR_034089	chr1 : 93775665 - 93811368	Down	TMED5 (-147232), DR1 (-17928)
LOC257396	NR_034107	chr5 : 52405686 - 52410952	Down	MOCS2 (-2730)
DNAJC27-AS1	NR_034113	chr2 : 25194980 - 25262563	Down	DNAJC27 (-33809), POMC (+163000)
LINC01270	NR_034124	chr20 : 48909256 - 48931456	Down	PTPN1 (-206535), CEBPB (+112980)
LINC00959	NR_034125	chr10 : 131862161 - 131909081	Down	EBF3 (-123516), GLRX3 (-49042)
LINC01160	NR_034126	chr1 : 112141628 - 112150940	Down	ADORA3 (-100174), RAP1A (-16121)
LINC00939	NR_034132	chr12 : 126443233 - 126467920	Down	TMEM132B (+644415)
LINC01530	NR_034159	chr19 : 52095035 - 52097633	Down	ENSG00000167765 (+1299), ZNF175 (+21783)
LINC00997	NR_036501	chr7 : 32797897 - 32802536	Down	FKBP9 (-196800), AVL9 (+265116)
LOC728752	NR_036504	chr19 : 36980528 - 36981942	Down	ZNF566 (-772)
LOC100129917	NR_036511	chr4 : 773936 - 775636	Down	CPLX1 (+45200), PCGF3 (+75232)
LOC100289230	NR_036530	chr5 : 98264837 - 98266713	Down	CHD1 (-3535)
LOC644656	NR_036539	chr11 : 9481102 - 9482245	Down	ZNF143 (-838)
LINC01061	NR_037596	chr4 : 120326677 - 120331815	Down	FABP2 (-85701), PDE5A (+220900)
LHX4-AS1	NR_037642	chr1 : 180199432 - 180244188	Down	LHX4 (+22389), ACBD6 (+250279)
LINC01963	NR_037701	chr2 : 217081611 - 217084915	Down	XRCC5 (+111076), MARCH4 (+153487)
PCAT18	NR_024259	chr18 : 24267584 - 24283602	Down	KCTD1 (-146194), AQP4 (+170189)
SCARNA17	NR_003003	chr18 : 47340392 - 47340813	Down	ACAA2 (-273)
LOC100506730	NR_037847	chr1 : 19619740 - 19622230	Down	AKR7A3 (-5241), AKR7A2 (+17655)
LOC100505918	NR_037851	chr1 : 168369426 - 168391894	Down	TBX19 (+130382), XCL2 (+132575)
LOC728730	NR_037875	chr2 : 39664556 - 39828484	Down	TMEM178A (-146539), MAP4K3 (-82067)
TMCC1-AS1	NR_037893	chr3 : 129612713 - 129627755	Down	TRH (-72914), TMCC1 (-20925)
ARHGEF26-AS1	NR_037901	chr3 : 153742189 - 153975616	Down	ARHGEF26 (+20111), DHX36 (+183383)
LINC00482	NR_038080	chr17 : 79276623 - 79283048	Down	SLC38A10 (-10731), TMEM105 (+24638)
LINC00665	NR_038278	chr19 : 36803962 - 36822620	Down	ZNF565 (-107308), ZFP14 (+56810)
LINC01125	NR_038386	chr2 : 98286205 - 98319529	Down	ZAP70 (-27156), ACTR1B (-22297)
STPG3-AS1	NR_038389	chr9 : 140144670 - 140147949	Down	NELFB (-3315)
LOC284865	NR_038460	chr22 : 20186252 - 20192441	Down	RTN4R (+66707), ZDHHC8 (+69876)
LINC00672	NR_038847	chr17 : 37081420 - 37085637	Down	LASP1 (+57417), PLXDC1 (+224373)
LINC01426	NR_038885	chr21 : 36118121 - 36157168	Down	CLIC6 (+95957), RUNX1 (+283996)

LOC401320	NR_038889	chr7 : 30587972 - 30617395	Down	GGCT (-58224), GARS (-31613)
LINC00641	NR_038970	chr14 : 21668237 - 21675059	Down	OR5AU1 (-47426), HNRNPC (+65990)
RBM26-AS1	NR_038991	chr13 : 79980443 - 79998468	Down	NDFIP2 (-65831), RBM26 (-9533)
KCNQ1OT1	NR_002728	chr11 : 2466220 - 2870340	Down	KCNQ1 (+202059), CDKN1C (+238831)
LINC01089	NR_002809	chr12 : 122233172 - 122241390	Down	SETD1B (-5357), RHOF (-5113)
LOC286437	NR_039980	chrX : 103139054 - 103401708	Down	H2BFWT (-2122)
LINC01004	NR_039981	chr7 : 104622193 - 104631612	Down	KMT2E (-27723), LHFPL3 (+657799)
ERVK13-1	NR_040023	chr16 : 2708389 - 2723440	Down	KCTD5 (-16561), PDPK1 (+127950)
LOC157273	NR_040039	chr8 : 9182560 - 9192590	Down	TNKS (-225849), PPP1R3B (-179369)
SMG7-AS1	NR_040063	chr1 : 183430010 - 183441117	Down	NMNAT2 (-47827), SMG7 (-6074)
LINC00261	NR_001558	chr20 : 22541191 - 22559280	Down	FOXA2 (+14865), PAX1 (+863939)
ENTPD3-AS1	NR_040100	chr3 : 40428672 - 40494799	Down	RPL14 (-37095), ENTPD3 (+33089)
LINC00941	NR_040245	chr12 : 30948614 - 30955645	Down	TSPAN11 (-127762), CAPRIN2 (-44682)
PRICKLE2-AS1	NR_045697	chr3 : 64053639 - 64211131	Down	PSMD6 (-123149), PRICKLE2 (+78746)
LINC00864	NR_046091	chr10 : 89156330 - 89167457	Down	GLUD1 (-307271), MINPP1 (-102738)
LINC01146	NR_046094	chr14 : 88490893 - 88553688	Down	GPR65 (+50823), KCNK10 (+267302)
LOC284581	NR_046097	chr1 : 205831206 - 205865215	Down	PM20D1 (-28966), SLC26A9 (+64377)
MIRLET7DHG	NR_046163	chr9 : 96938851 - 96966848	Down	ZNF169 (-68743), PTPDC1 (+106104)
MGC27345	NR_046216	chr7 : 127937737 - 127947816	Down	RBM28 (+41185), LEP (+61440)
LOC171391	NR_046316	chr11 : 779453 - 780755	Down	PDDC1 (-2620)
LOC728040	NR_046377	chr4 : 74374519 - 74394250	Down	AFM (+36985), RASSF6 (+101963)
ZNRF3-AS1	NR_046851	chr22 : 29279754 - 29453476	Down	KREMEN1 (-102491), ZNRF3 (+87035)
UGDH-AS1	NR_047679	chr4 : 39529458 - 39640481	Down	UGDH (-55732), SMIM14 (+55740)
LOC100506688	NM_001242737	chr5 : 988424 - 997455	Down	BRD9 (-100001), NKD2 (-16004)
PTCSC3	NR_049735	chr14 : 36539632 - 36645857	Down	MBIP (+197137), BRMS1L (+297221)

Plus (+) means upstream while minus (-) means downstream.

Two genes omitted since chromosome location not well-established.

Table S5. LncRNAs that were differentially expressed after primary human hepatocytes were treated with 100 µM DEET plus 10 µM Fipronil.

Differentially Expressed lncRNAs (100 µM DEET plus 10 µM Fipronil mixture)

Gene symbol	Genbank Accession	Chromosome Coordinates	Gene Expression	LncRNA Associated Genes
RPL23AP7	NR_000029	chr2 : 114368815 - 114384715	Up	RABL2A (-8041), FOXD4L1 (+120104)
CYP2B7P	NR_001278	chr19 : 41430169 - 41456565	Up	CYP2A7 (-54710), CYP2B6 (-53837)
MT1L	NR_001447	chr16 : 56651372 - 56652730	Up	MT1E (-7336), MT2A (+9940)
SMG1P5	NR_002453	chr16 : 30278913 - 30346695	Up	NPIPB13 (-55682), CD2BP2 (+53878)
TEKT4P2	NR_038327	chr21 : 9907188 - 9968593	Up	NONE
STAG3L4	NR_040585	chr7 : 66767624 - 66786513	Up	TYW1 (+315267)
CIDECP	NR_002786	chr3 : 10059236 - 10067820	Up	FANCD2 (-4570)
MGC72080	NR_002822	chr7 : 97595907 - 97601638	Up	ASNS (-96919), OCM2 (+20735)
SNX29P2	NR_002939	chr16 : 29313607 - 29376380	Up	NPIPB11 (+70356), LAT (+348847)
GBP1P1	NR_003133	chr1 : 89873237 - 89890493	Up	LRRC8B (-108597), GBP6 (+52248)
RP9P	NR_003500	chr7 : 32956426 - 32982782	Up	FKBP9 (-27413), AVL9 (+434503)
GTF2IP4	NR_003580	chr7 : 72569011 - 72621336	Up	STAG3L3 (-118930), NSUN5 (+127639)
LOC728554	NR_003615	chr5 : 177302261 - 177311269	Up	PROP1 (+116478), B4GALT7 (+279664)
WASH3P	NR_003659	chr15 : 102501015 - 102519296	Up	OR4F4 (-46858)
WASH2P	NR_024077	chr2 : 114341229 - 114361294	Up	RABL2A (-33544), FOXD4L1 (+94601)
RASA4CP	NR_024116	chr7 : 44068485 - 44083895	Up	DBNL (-8120), UBE2D4 (+110147)
FAM86DP	NR_024241	chr3 : 75470702 - 75484266	Up	CNTN3 (-907193), FRG2C (-235997)
FAM86FP	NR_024254	chr12 : 8383644 - 8395542	Up	CLEC6A (-218929), FAM90A1 (-9379)
MSL3P1	NR_024322	chr2 : 234774089 - 234777055	Up	TRPM8 (-50471), HJURP (-12360)
FBLL1	NR_024356	chr5 : 167956581 - 167957639	Up	FBLL1 (+989)
RPSAP58	NR_003662	chr19 : 23945815 - 24010919	Up	ZNF726 (-119325), RPSAP58 (+32551)
FGF7P6	NR_003674	chr9 : 46687556 - 46746820	Up	NONE
RPL23AP82	NR_026981	chr22 : 51195513 - 51238065	Up	RABL2B (+5281), ACR (+40165)
GOLGA2P7	NR_027001	chr15 : 84860599 - 84898920	Up	ZSCAN2 (-264476), ADAMTSL3 (+556922)
ROCK1P1	NR_033770	chr18 : 109064 - 122222	Up	TUBB8P12 (-66086), USP14 (-42965)
SERPINB9P1	NR_033851	chr6 : 2854890 - 2876744	Up	SERPINB1 (-23577), SERPINB9 (+37697)
LOC654342	NR_027238	chr2 : 91824708 - 91847975	Up	NONE

PMS2P4	NR_022007	chr7 : 66741117 - 66767429	Up	TYW1 (+292471)
TREML3P	NR_027256	chr6 : 41176291 - 41185685	Up	TREML4 (-15074), TREML2 (-12056)
BMS1P20	NR_027293	chr22 : 22652462 - 22677324	Up	VPREB1 (+65806), ZNF280B (+198612)
PI4KAP2	NR_003700	chr22 : 21827286 - 21871780	Up	UBE2L3 (-54203), HIC2 (+52784)
FKBP9P1	NR_003949	chr7 : 55748766 - 55772260	Up	VOPP1 (-120295), SEPT14 (+169969)
EPB41L4A-AS1	NR_015370	chr5 : 111496222 - 111498198	Up	NREP (-184582), EPB41L4A (+257803)
UCA1	NR_015379	chr19 : 15939756 - 15947131	Up	OR10H1 (-24508), CYP4F2 (+65486)
MIR4435-2HG	NR_015395	chr2 : 112124590 - 112252692	Up	BCL2L11 (+310135), ANAPC1 (+453626)
ZMIZ1-AS1	NR_015429	chr10 : 80703082 - 80827205	Up	ZMIZ1 (-63648), RPS24 (+971626)
UBA6-AS1	NR_015439	chr4 : 68566995 - 68588222	Up	UBA6 (-10712), GNRHR (+42469)
LINC00998	NR_015442	chr7 : 112756772 - 112758637	Up	LINC00998 (+963)
DANCR	NR_024031	chr4 : 53578620 - 53580305	Up	USP46 (-53961), ERVMER34-1 (+38344)
NCBP2-AS2	NR_024388	chr3 : 196669493 - 196670884	Up	NCBP2 (-721)
LINC00467	NR_026761	chr1 : 211556096 - 211605877	Up	TRAF5 (+80808), RD3 (+85272)
SVIL-AS1	NR_003930	chr10 : 29698500 - 29711299	Up	LYZL1 (+126910), SVIL (+319830)
URB1-AS1	NR_026845	chr21 : 33765441 - 33766266	Up	URB1 (-519)
MGC12916	NR_026880	chr17 : 14204505 - 14249492	Up	HS3ST3B1 (+22599), PMP22 (+938907)
DRAIC	NR_026979	chr15 : 69854058 - 69863779	Up	RPLP1 (+113796), TLE3 (+531596)
PRR34-AS1	NR_027034	chr22 : 46446338 - 46454402	Up	PPARA (-96129), WNT7B (-77361)
LINC00116	NR_027063	chr2 : 110969105 - 110980517	Up	NPHP1 (-12192), LIMS4 (+255582)
LOC153910	NR_027311	chr6 : 142847591 - 142959026	Up	GPR126 (+280243), HIVEP2 (+363029)
LINC00863	NR_029408	chr10 : 88998423 - 89103331	Up	MINPP1 (-213755), GLUD1 (-196254)
LOC284344	NR_033888	chr19 : 43715942 - 43752798	Up	PSG4 (-24444), PSG9 (+39310)
LINC00673	NR_036488	chr17 : 70399462 - 70588943	Up	SOX9 (+377042), SLC39A11 (+594624)
LOC100294145	NR_037177	chr6 : 32861952 - 32871535	Up	HLA-DMB (+42103), PSMB9 (+44806)
SNHG16	NR_038108	chr17 : 74553845 - 74561430	Up	PRCD (+21466), ST6GALNAC2 (+24572)
LOC100506844	NR_038269	chr12 : 58325231 - 58329947	Up	CTDSP2 (-87067), XRCC6BP1 (-7771)
SNHG9	NR_003142	chr16 : 2014996 - 2015505	Up	RNF151 (-1624), RPS2 (-390)
SNHG5	NR_003038	chr6 : 86386724 - 86388451	Up	SYNCRIP (-34585)
LINC00888	NR_038301	chr3 : 183165395 - 183173800	Up	MCF2L2 (-23535), KLHL6 (+103879)
HCG26	NR_002812	chr6 : 31439005 - 31440185	Up	MICB (-26297), MICA (+68239)

CYTOR	NR_024204	chr2 : 87754973 - 87821030	Up	PLGLB1 (-539027), PLGLB2 (-259604)
OSER1-AS1	NR_038337	chr20 : 42839599 - 42854667	Up	JPH2 (-30915), FITM2 (+92676)
LOC100507389	NR_038455	chr3 : 142645516 - 142661378	Up	PCOLCE2 (-45402), PAQR9 (+28731)
SNHG8	NR_003584	chr4 : 119199916 - 119200978	Up	PRSS12 (+73711), NDST3 (+244947)
ZFAS1	NR_003604	chr20 : 47862438 - 47905795	Up	ZNFX1 (+10639), DDX27 (+48233)
SNHG15	NR_003697	chr7 : 45022626 - 45026259	Up	CCM2 (-42182), MYO1G (-5746)
GAS5	NR_002578	chr1 : 173832385 - 173837125	Up	ZBTB37 (-2738)
SNHG6	NR_002599	chr8 : 67782983 - 67837777	Up	MCMDC2 (+27396), TCF24 (+64445)
LYPLAL1-AS1	NR_038845	chr1 : 219254316 - 219347130	Up	LYPLAL1 (-46463), TGFB2 (+781146)
HCP5	NR_040662	chr6 : 31430956 - 31433586	Up	MICB (-33621), MICA (+60915)
HEIH	NR_045680	chr5 : 180256953 - 180262726	Up	MGAT1 (-23011), ZFP62 (+28437)
H19	NR_002196	chr11 : 2016405 - 2019065	Up	MRPL23 (+49227), IGF2 (+144733)
SH3BP5-AS1	NR_046084	chr3 : 15295690 - 15382901	Up	SH3BP5 (+34840), CAPN7 (+91546)
LINC01619	NM_001256373	chr12 : 92378751 - 92539673	Up	DCN (-882312), BTG1 (+80461)
LINC01587	NM_005750	chr4 : 5526882 - 5529527	Up	C4orf6 (+1322), EVC2 (+182089)
EXOC3-AS1	NM_138464	chr5 : 441642 - 443258	Up	EXOC3 (-823), C5orf55 (+808)
RCHY1	NM_001008925	chr4 : 76404246 - 76439640	Up	THAP6 (-17740), PARM1 (+563617)
HLA-DRB6	NR_001298	chr6 : 32520489 - 32527779	Down	HLA-DRB5 (-26070), HLA-DRB1 (+33491)
HLA-H	NR_001434	chr6 : 29855382 - 29858856	Down	HLA-A (-51918), HLA-G (+62363)
TPTEP1	NR_001591	chr22 : 17082800 - 17129720	Down	CCT8L2 (-32560), XKR3 (+196329)
CMAHP	NR_002174	chr6 : 25081294 - 25138620	Down	FAM65B (-198762), LRRC16A (-169699)
PGM5P2	NR_002836	chr9 : 69080243 - 69147854	Down	FOXD4L6 (+88155)
SCART1	NR_002934	chr10 : 135267431 - 135281953	Down	CYP2E1 (-59218), MTG1 (+67017)
RPL32P3	NR_003111	chr3 : 129101676 - 129118282	Down	H1FX (-74859), EFCAB12 (+37515)
LOC220729	NR_003266	chr3 : 197340897 - 197354752	Down	BDH1 (-64967), KIAA0226 (+115765)
CES1P1	NR_003276	chr16 : 55794510 - 55808826	Down	CES1 (+65402), SLC6A2 (+111112)
EP400NL	NR_003290	chr12 : 132568827 - 132610885	Down	DDX51 (+39024), EP400 (+155348)
GUSBP2	NR_003504	chr6 : 26839265 - 26924333	Down	ZNF322 (-221819), HIST1H2BJ (+218730)
AFG3L1P	NR_003226	chr16 : 90036182 - 90067195	Down	CENPBD1 (-12747), DBNDD1 (+24840)
ZNF37BP	NR_026777	chr10 : 43008960 - 43048280	Down	ZNF33B (+105372)
PFN1P2	NR_003242	chr1 : 144610814 - 144612727	Down	PPIAL4B (-247525), NBPF9 (-199977)

LOC646214	NR_027053	chr15 : 21932513 - 21940739	Down	OR4M2 (-431852)
LRRC37A4P	NR_002940	chr17 : 43583248 - 43597889	Down	CRHR1 (-271342), PLEKHM1 (-22459)
HERC2P4	100289574	chr16 : 32162608 - 32163874	Down	ZNF267 (+278162), TP53TG3 (+524812)
DPY19L2P2	NR_003561	chr7 : 102815461 - 102920913	Down	NAPEPLD (-78558), PMPCB (-69682)
PI4KAP1	NR_003563	chr22 : 20383730 - 20398695	Down	GGTLC3 (-23416), USP41 (+353835)
ABCC6P1	NR_003569	chr16 : 18582569 - 18609607	Down	NOMO2 (-22660), RPS15A (+205568)
PDXDC2P	NR_003610	chr16 : 70010201 - 70099851	Down	PDPR (-92503), CLEC18A (+69943)
ARHGAP27P1-BPTFP1-KPNA2P3	NR_026899	chr17 : 62745779 - 62778117	Down	SMURF2 (-103762), LRRC37A3 (+152955)
LOC202181	NR_026921	chr5 : 177045500 - 177099278	Down	B4GALT7 (+45288), PROP1 (+350854)
UBE2Q2P1	NR_003661	chr15 : 85070426 - 85123412	Down	ZSCAN2 (-47317), ADAMTSL3 (+774081)
CROCCP3	NR_023386	chr1 : 16793930 - 16819196	Down	NECAP2 (+39332), NBPF1 (+133419)
CLUHP3	NR_024034	chr16 : 31711933 - 31718745	Down	ZNF720 (-9216), AHSP (+176154)
AKR1C8P	NR_027916	chr10 : 5196654 - 5227150	Down	AKR1CL1 (+15194), AKR1C3 (+75917)
ANKRD20A9P	NR_027995	chr13 : 19408542 - 19446109	Down	TUBA3C (+328666)
PMS2P9	NR_028058	chr7 : 76668796 - 76682355	Down	POMZP3 (-419018), FGL2 (+153567)
SMA4	NR_024054	chr5 : 69423288 - 69586004	Down	SERF1A (-691868), SMN2 (+159207)
GOLGA8IP	NR_024074	chr15 : 23255241 - 23262743	Down	GOLGA8S (-341001), GOLGA8I (+3750)
SPDYE7P	NR_003666	chr7 : 72333317 - 72339655	Down	CALN1 (-459128), POM121 (-13450)
FAHD2CP	NR_003698	chr2 : 96676298 - 96700727	Down	ANKRD36C (-30972), GPAT2 (+13209)
PPIEL	NR_003929	chr1 : 39957317 - 40025370	Down	BMP8A (+34026), PABPC4 (+50729)
AQP7P1	NR_002817	chr9 : 67270214 - 67289492	Down	ANKRD20A1 (-646908)
HLA-J	NR_024240	chr6 : 29968787 - 30028961	Down	ZNRD1 (-30157), HLA-A (+89837)
ANKRD36BP1	NR_026844	chr1 : 168214818 - 168216668	Down	TBX19 (-34535), SFT2D2 (+20497)
APOC1P1	NR_028412	chr19 : 45430059 - 45434643	Down	APOC4 (-13144), APOC1 (+14847)
MTMR9LP	NR_026850	chr1 : 32697260 - 32707311	Down	LCK (-14554), EIF3I (+14315)
GOLGA2P5	NR_024261	chr12 : 100550174 - 100567121	Down	ANKS1B (-180633), ACTR6 (-35217)
ESPNP	NR_026567	chr1 : 17017712 - 17046652	Down	CROCC (-216263), NBPF1 (-92200)
WHAMMP2	NR_026589	chr15 : 28982728 - 29003508	Down	APBA2 (-138002), GOLGA8M (-35649)
BMS1P4	NR_026592	chr10 : 75458908 - 75490272	Down	SEC24C (-29559), AGAP5 (-17036)

RPS10P7	NR_026667	chr1 : 201489031 - 201489720	Down	ENSG00000269690_no gene symbol (-102637), CSRP1 (-10792)
CROCCP2	NR_026752	chr1 : 16944750 - 16957401	Down	CROCC (-297369), NBPF1 (-11094)
LOC728989	NR_024442	chr1 : 146490894 - 146514599	Down	NBPF12 (+128691), PRKAB2 (+141376)
MST1P2	NR_027504	chr1 : 16972068 - 16976915	Down	CROCC (-273953), NBPF1 (-34510)
BEND3P3	NR_027512	chr10 : 81442730 - 81448650	Down	SFTPA1 (+74989), SFTPD (+263169)
FAM86B3P	NR_024361	chr8 : 8086091 - 8102387	Down	SGK223 (+149769), ZNF705B (+293095)
FAM45BP	NR_027141	chrX : 129628914 - 129631421	Down	RBMX2 (+94225), ENOX2 (+407040)
SEPT7P2	NR_024271	chr7 : 45763385 - 45808617	Down	IGFBP1 (-141955), ADCY1 (+171880)
FAM86EP	NR_024253	chr4 : 3943668 - 3957148	Down	ADRA2C (+182283), OTOP1 (+278208)
CEACAM22P	NR_027754	chr19 : 45041044 - 45060150	Down	IGSF23 (-66343), ZNF180 (-46023)
LOC155060	NR_036573	chr7 : 148982371 - 148994403	Down	ZNF783 (+29125), ZNF777 (+169827)
ADAM1A	NR_036636	chr12 : 112336866 - 112339706	Down	MAPKAPK5 (+57856), TMEM116 (+112629)
CCDC144B	NR_036647	chr17 : 18441114 - 18528930	Down	TBC1D28 (+60715), LGALS9C (+104934)
MT1JP	NR_036677	chr16 : 56669650 - 56670998	Down	MT1A (-2254)
AZGP1P1	NR_036679	chr7 : 99578384 - 99581860	Down	ZKSCAN1 (-33082), AZGP1 (-6342)
LOC730102	NR_037167	chr1 : 177975274 - 178007142	Down	RASAL2 (-72068), SEC16B (-52158)
RPL23AP87	NR_029406	chr17 : 81174665 - 81188573	Down	METRNL (+144052)
BTN2A3P	NR_027795	chr6 : 26421618 - 26430816	Down	BTN3A3 (-14483), BTN3A1 (+23752)
FAM153C	NR_038353	chr5 : 177433966 - 177476088	Down	N4BP3 (-85417), PROP1 (-31784)
YY1P2	NR_033658	chr2 : 139654893 - 139656744	Down	NXPH2 (-117901)
DNM1P41	NR_033787	chr15 : 85045805 - 85060078	Down	ZSCAN2 (-91294), ADAMTSL3 (+730104)
HLA-L	NR_027822	chr6 : 30201815 - 30314635	Down	TRIM26 (-77082), TRIM39 (-36396)
AKR7L	NR_040288	chr1 : 19592475 - 19600568	Down	EMC1 (-18476), AKR7A3 (+19222)
ZNF767P	NR_027788	chr7 : 149244244 - 149321881	Down	KRBA1 (-129085), ZNF746 (-88165)
GOLGA8T	NR_033933	chr15 : 30427989 - 30439395	Down	GOLGA8T (+6340), CHRFAM7A (+252060)
CTSLP2	NR_033407	chr10 : 48155942 - 48158691	Down	ASAH2C (-102299), AGAP9 (-58519)
GTF2H2B	NR_033417	chr5 : 69711196 - 69746189	Down	SERF1A (-467821), SMN2 (+383254)
HERC2P7	NR_036470	chr15 : 23390721 - 23393943	Down	GOLGA8S (-207661), GOLGA8I (+137090)
TMEM198B	NR_036476	chr12 : 56211805 - 56236767	Down	DNAJC14 (+279)
HTATSF1P2	NR_033884	chr6 : 3020389 - 3025005	Down	RIPK1 (-45961), NQO2 (+22489)

LOC100130075	NR_073494	chr12 : 69198216 - 69199274	Down	MDM2 (-3211)
GCSHP3	NR_033248	chr2 : 206980296 - 206981296	Down	INO80D (-29890), NDUFS1 (+43122)
WASH5P	NR_033266	chr19 : 60950 - 70966	Down	OR4F17 (-41513)
GLUD1P3	NR_048575	chr10 : 75491298 - 75495367	Down	AGAP5 (-35779), SEC24C (-10816)
ANKRD20A12P	NR_046228	chr1 : 142697420 - 142713605	Down	NONE
GOLGA8S	NR_038843	chr15 : 23599894 - 23613471	Down	MKRN3 (-203771), GOLGA8S (+6690)
LOC100132057	NR_033189	chr1 : 143687129 - 143714180	Down	PPIAL4G (+67226)
GUSBP11	NR_024448	chr22 : 23980674 - 24059610	Down	IGLL1 (-97647), RGL4 (-12906)
ALG1L9P	NR_073386	chr11 : 71498556 - 71524905	Down	KRTAP5-11 (-217810), DEFB108B (-32515)
PRKXP1	NR_073405	chr15 : 101087956 - 101099488	Down	ASB7 (-49017), CERS3 (-8797)
SMG1P7	NR_033959	chr16 : 70253483 - 70259936	Down	EXOSC6 (+29123), CLEC18C (+48782)
GUSBP9	NR_033968	chr5 : 69776869 - 69881549	Down	SERF1A (-367305), SMN2 (+483770)
FRG1HP	NR_034006	chr9 : 68726540 - 68748372	Down	FOXD4L6 (+464748), ANKRD20A1 (+810695)
LOC100131257	NR_034022	chr7 : 7115400 - 7136417	Down	CCZ1B (-259508), C1GALT1 (-96337)
FRG1JP	NR_033907	chr9 : 68427782 - 68454375	Down	ANKRD20A1 (+514318), FOXD4L6 (+761125)
URAHP	NR_027335	chr16 : 90089007 - 90114191	Down	C16orf3 (-5290), URAHP (+12582)
GUSBP3	NR_027386	chr5 : 68935289 - 69006272	Down	SERF1B (-350315), GTF2H2C (+114712)
PRORSD1P	NR_027258	chr2 : 55509454 - 55511607	Down	MTIF2 (-14216), CCDC88A (+136218)
C3P1	NR_027300	chr19 : 10152031 - 10184813	Down	RDH8 (+44497), ANGPTL6 (+45050)
LOC652276	NR_015441	chr16 : 2653384 - 2680495	Down	KCTD5 (-65536), PDPK1 (+78975)
DKFZP586I1420	NR_002186	chr7 : 30409665 - 30412410	Down	ZNRF2 (+87115), NOD1 (+107358)
MBL1P	NR_002724	chr10 : 81664653 - 81691557	Down	SFTPD (+30754), SFTPA1 (+307404)
KCNQ1OT1	NR_002728	chr11 : 2466220 - 2870340	Down	KCNQ1 (+202059), CDKN1C (+238831)
MEG3	NR_002766	chr14 : 101292444 - 101327360	Down	RTL1 (+41282), DLK1 (+116738)
DIO3OS	NR_002770	chr14 : 102018559 - 102022013	Down	DIO3 (-7402), LOC100288160 (+661021)
LINC01089	NR_002809	chr12 : 122233172 - 122241390	Down	SETD1B (-5357), RHOF (-5113)
MALAT1	NR_002819	chr11 : 65265232 - 65273939	Down	SCYL1 (-22962), FRMD8 (+115516)
LINC00261	NR_001558	chr20 : 22541191 - 22559280	Down	FOXA2 (+14865), PAX1 (+863939)
XIST	NR_001564	chrX : 73012039 - 73072588	Down	ZCCHC13 (-481711), CHIC1 (+259270)
KC6	NR_002838	chr18 : 39060235 - 39100561	Down	PIK3C3 (-454773)
SCARNA17	NR_003003	chr18 : 47340392 - 47340813	Down	ACAA2 (-273)

TSIX	NR_003255	chrX : 73012039 - 73072588	Down	ZCCHC13 (-481711), CHIC1 (+259270)
CDKN2B-AS1	NR_003529	chr9 : 21994789 - 22121093	Down	DMRTA1 (-388899), CDKN2B (-48579)
LOC441204	NR_015364	chr7 : 26443107 - 26535986	Down	SNX10 (+158004), SKAP2 (+414815)
A1BG-AS1	NR_015380	chr19 : 58858171 - 58874214	Down	A1BG (-1328)
LINC00294	NR_015451	chr11 : 33097695 - 33101000	Down	TCP11L1 (+38385), CSTF3 (+83663)
SYNE3	NR_026779	chr14 : 95873603 - 95876427	Down	CLMN (-88772), SYNE3 (+67158)
PSMG3-AS1	NR_021487	chr7 : 1609708 - 1629261	Down	ELFN1 (-108270), TMEM184A (-23419)
LINC00923	NR_024172	chr15 : 98285845 - 98417659	Down	ARRDC4 (-152176)
LOC100132111	NR_024237	chr1 : 151810338 - 151816641	Down	RORC (-9142), THEM5 (+12683)
PCAT18	NR_024259	chr18 : 24267584 - 24283602	Down	KCTD1 (-146194), AQP4 (+170189)
LINC00115	NR_024321	chr1 : 761585 - 762902	Down	OR4F16 (-140191), SAMD11 (-98874)
HNF1A-AS1	NR_024345	chr12 : 121407640 - 121410095	Down	SPPL3 (-66694), HNF1A (-7478)
LINC01000	NR_024368	chr7 : 128281294 - 128301052	Down	CALU (-88173), METTL2B (+174390)
PSMD5-AS1	NR_024408	chr9 : 123605319 - 123616651	Down	PSMD5 (-5723), PHF19 (+28621)
LINC01018	NR_024423	chr5 : 6582286 - 6588613	Down	NSUN2 (+47954), UBE2QL1 (+136714)
PTGES2-AS1	NR_024425	chr9 : 130890807 - 130892913	Down	PTGES2 (-1119)
MIR100HG	NR_024430	chr11 : 121959810 - 122073770	Down	UBASH3B (-509593), BLID (-29867)
LINC00926	NR_024433	chr15 : 57592562 - 57599967	Down	CGNL1 (-72438), TCF12 (+385444)
LOC100128288	NR_024447	chr17 : 8261730 - 8263859	Down	KRBA2 (+12063), ODF4 (+19613)
NDUFB2-AS1	NR_024454	chr7 : 140395135 - 140406446	Down	NDUFB2 (+4320), BRAF (+223773)
LINC01134	NR_024455	chr1 : 3816967 - 3832011	Down	AJAP1 (-890616), C1orf174 (-7640)
LOC100190986	NR_024456	chr16 : 21443344 - 21445776	Down	METTL9 (-166237), NPIPB3 (-13482)
RAMP2-AS1	NR_024461	chr17 : 40905946 - 40915060	Down	RAMP2 (-2709)
HECTD2-AS1	NR_024467	chr10 : 93066718 - 93371217	Down	HECTD2 (+48866), PPP1R3C (+173843)
MRPL23-AS1	NR_024471	chr11 : 2004438 - 2011150	Down	MRPL23 (+39286), IGF2 (+154674)
LOC100128573	NR_024491	chr19 : 7537722 - 7538247	Down	PEX11G (+15920), ARHGEF18 (+33411)
FLJ42627	NR_024492	chr16 : 2688982 - 2696130	Down	KCTD5 (-39920), PDPK1 (+104591)
LINC00999	NR_024497	chr10 : 38717073 - 38741081	Down	ZNF37A (+345813)
KMT2E-AS1	NR_024586	chr7 : 104650988 - 104654588	Down	KMT2E (-1838)
LINC02035	NR_024618	chr3 : 122605359 - 122611263	Down	SEMA5B (+86670), DIRC2 (+94669)
MIR600HG	NR_026677	chr9 : 125871772 - 125877756	Down	GPR21 (+77958), STRBP (+156091)

LINC01558	NR_026773	chr6 : 168185218 - 168197539	Down	TCP10 (-393425), C6orf123 (+6160)
LINC00240	NR_026775	chr6 : 26924771 - 26991753	Down	ZNF322 (-298282), HIST1H2BJ (+142267)
LINC00574	NR_026780	chr6 : 170188885 - 170202969	Down	C6orf70 (+44206), DLL1 (+403634)
HCG27	NR_026791	chr6 : 31165536 - 31171745	Down	POU5F1 (-30171), HLA-C (+71222)
PSORS1C3	NR_026816	chr6 : 31141511 - 31145676	Down	POU5F1 (-5124), HLA-C (+96269)
LINC00839	NR_026827	chr10 : 42970938 - 42990785	Down	ZNF33B (+153130)
BAIAP2-AS1	NR_026857	chr17 : 79002932 - 79008519	Down	BAIAP2 (-3236)
LINC00174	NR_026873	chr7 : 65841030 - 65865395	Down	KCTD7 (-352430), TPST1 (+183027)
PDCD4-AS1	NR_026932	chr10 : 112628647 - 112630662	Down	PDCD4 (-1910)
LINC01554	NR_026936	chr5 : 95187935 - 95195836	Down	GLRX (-33468), ELL2 (+105889)
LINC00663	NR_026956	chr19 : 19867180 - 19887222	Down	ZNF14 (-33295), ZNF506 (+55359)
LOC100130691	NR_026966	chr2 : 178148235 - 178257419	Down	NFE2L2 (-72968), AGPS (-54545)
LOC143666	NR_026967	chr11 : 573807 - 575885	Down	PHRF1 (-1675)
LINC01140	NR_026985	chr1 : 87595447 - 87634886	Down	LMO4 (-178984), RP5-1052I5.2 (+156475)
LINC00265	NR_026999	chr7 : 39773166 - 39834222	Down	CDK13 (-185942), RALA (+140612)
LOC93429	NR_027003	chr19 : 46713498 - 46718094	Down	IGFL1 (-17213), IGFL2 (+64757)
THUMPD3-AS1	NR_027007	chr3 : 9430536 - 9439174	Down	THUMPD3 (+30119), LHFPL4 (+160631)
FAM41C	NR_027055	chr1 : 803450 - 812182	Down	OR4F16 (-185763), SAMD11 (-53302)
ESRG	NR_027122	chr3 : 54156692 - 55108584	Down	LRTM1 (+329463), CACNA2D3 (+475945)
LINC01126	NR_027251	chr2 : 43454349 - 43455994	Down	ZFP36L2 (-1424)
LINC00671	NR_027254	chr17 : 41026690 - 41050751	Down	G6PC (-14094), AOC3 (+35520)
MGC27382	NR_027310	chr1 : 78695282 - 78835147	Down	PTGFR (-191542), GIPC2 (+253629)
MZF1-AS1	NR_027334	chr19 : 59070552 - 59086164	Down	UBE2M (-8031), MZF1 (+6584)
LINC00910	NR_027412	chr17 : 41447212 - 41466266	Down	ARL4D (-19588), TMEM106A (+92845)
LOC100272217	NR_027440	chr9 : 133452736 - 133454881	Down	FUBP3 (-1184)
MAN1B1-AS1	NR_027447	chr9 : 139979397 - 139981269	Down	MAN1B1 (-1046)
LINC00893	NR_027455	chrX : 148609131 - 148621312	Down	IDS (-28345), CXorf40A (-6965)
LINC00894	NR_027456	chrX : 149106765 - 149185018	Down	MAMLD1 (-385794), MAGEA8 (+135951)
TAPT1-AS1	NR_027696	chr4 : 16228285 - 16259810	Down	TAPT1 (-15884), LDB2 (+656384)
MIR99AHG	NR_027790	chr21 : 17442841 - 17982094	Down	USP25 (+610124)
AFDN-AS1	NR_027906	chr6 : 168224569 - 168227476	Down	MLLT4 (-1648)

NEAT1	NR_028272	chr11 : 65190268 - 65194003	Down	SCYL1 (-100412), FRMD8 (+38066)
FTX	NR_028379	chrX : 73164158 - 73513409	Down	ZCCHC13 (-185241), CHIC1 (+555740)
ADORA2A-AS1	NR_028483	chr22 : 24823529 - 24890783	Down	UPB1 (-33872), ADORA2A (+33626)
LOC100289511	NR_029378	chr14 : 70232999 - 70238722	Down	SLC10A1 (+28145), SRSF5 (+42244)
LINC01347	NR_029401	chr1 : 243219615 - 243265046	Down	PLD5 (-554511), CEP170 (+176021)
LOC643406	NR_029405	chr20 : 5451841 - 5457780	Down	PROKR2 (-157433), GPCPD1 (+136861)
TLR8-AS1	NR_030727	chrX : 12920935 - 12961419	Down	TMSB4X (-52052), TLR8 (+16438)
SLC25A21-AS1	NR_033240	chr14 : 37147125 - 37642998	Down	SLC25A21 (+247009), PAX9 (+263992)
SLC25A25-AS1	NR_033374	chr9 : 130873449 - 130881013	Down	PTGES2-AS1 (-13381), SLC25A25 (+46751)
LOC100422737	NR_033557	chr6 : 107165326 - 107235300	Down	C6orf203 (-149094), QRSL1 (+122860)
DKFZP434I0714	NR_033797	chr4 : 153457415 - 153460415	Down	FBXW7 (-1662)
FAM83H-AS1	NR_033849	chr8 : 144816309 - 144828507	Down	FAM83H (-6437), SCRIB (+75141)
LOC283177	NR_033852	chr11 : 134306375 - 134375555	Down	B3GAT1 (-78718)
LINC00514	NR_033861	chr16 : 3039054 - 3044510	Down	CLDN9 (-20675), PKMYT1 (-11242)
LINC01252	NR_033890	chr12 : 11700963 - 11717335	Down	PRB2 (-160650), ETV6 (-93639)
F11-AS1	NR_033900	chr4 : 187187117 - 187422212	Down	F11 (+117566), MTNR1A (+172056)
LINC00921	NR_033904	chr16 : 3313767 - 3317566	Down	ZNF263 (-17276), MEFV (-9040)
LOC100288069	NR_033908	chr1 : 700244 - 714068	Down	SAMD11 (-153962), OR4F16 (-85103)
LOC100132077	NR_033937	chr9 : 97094757 - 97123230	Down	HIATL1 (-27839), ZNF169 (+87401)
LINC00842	NR_033957	chr10 : 47096453 - 47151400	Down	NPY4R (+40393), ANXA8L1 (+50113)
LINC00885	NR_034088	chr3 : 195869506 - 195887761	Down	TFRC (-69574), ZDHHC19 (+59632)
CCDC18-AS1	NR_034089	chr1 : 93775665 - 93811368	Down	TMED5 (-147232), DR1 (-17928)
DNAJC27-AS1	NR_034113	chr2 : 25194980 - 25262563	Down	DNAJC27 (-33809), POMC (+163000)
NDUFA6-AS1	NR_034118	chr22 : 42486936 - 42521354	Down	NDUFA6 (-17186), CYP2D6 (+22763)
LINC01270	NR_034124	chr20 : 48909256 - 48931456	Down	PTPN1 (-206535), CEBPB (+112980)
LINC00959	NR_034125	chr10 : 131862161 - 131909081	Down	EBF3 (-123516), GLRX3 (-49042)
LINC01160	NR_034126	chr1 : 112141628 - 112150940	Down	ADORA3 (-100174), RAP1A (-16121)
LINC00939	NR_034132	chr12 : 126443233 - 126467920	Down	TMEM132B (+644415)
LINC01530	NR_034159	chr19 : 52095035 - 52097633	Down	AC018755.1 (+1299), ZNF175 (+21783)
LINC00997	NR_036501	chr7 : 32797897 - 32802536	Down	FKBP9 (-196800), AVL9 (+265116)
LOC728752	NR_036504	chr19 : 36980528 - 36981942	Down	ZNF566 (-772)

LOC100129917	NR_036511	chr4 : 773936 - 775636	Down	CPLX1 (+45200), PCGF3 (+75232)
LOC100289230	NR_036530	chr5 : 98264837 - 98266713	Down	CHD1 (-3535)
LOC644656	NR_036539	chr11 : 9481102 - 9482245	Down	ZNF143 (-838)
LINC01061	NR_037596	chr4 : 120326677 - 120331815	Down	FABP2 (-85701), PDE5A (+220900)
LHX4-AS1	NR_037642	chr1 : 180199432 - 180244188	Down	LHX4 (+22389), ACBD6 (+250279)
LINC01963	NR_037701	chr2 : 217081611 - 217084915	Down	XRCC5 (+111076), MARCH4 (+153487)
LOC100506730	NR_037847	chr1 : 19619740 - 19622230	Down	AKR7A3 (-5241), AKR7A2 (+17655)
LOC100505918	NR_037851	chr1 : 168369426 - 168391894	Down	TBX19 (+130382), XCL2 (+132575)
LOC653160	NR_037869	chr1 : 35441299 - 35444307	Down	DLGAP3 (-47617), ZMYM6NB (+8151)
LOC728730	NR_037875	chr2 : 39664556 - 39828484	Down	TMEM178A (-146539), MAP4K3 (-82067)
TMCC1-AS1	NR_037893	chr3 : 129612713 - 129627755	Down	TRH (-72914), TMCC1 (-20925)
LINC00482	NR_038080	chr17 : 79276623 - 79283048	Down	SLC38A10 (-10731), TMEM105 (+24638)
PITRM1-AS1	NR_038284	chr10 : 3179918 - 3215033	Down	PITRM1 (+17527), PFKP (+87764)
LINC01125	NR_038386	chr2 : 98286205 - 98319529	Down	ZAP70 (-27156), ACTR1B (-22297)
STPG3-AS1	NR_038389	chr9 : 140144670 - 140147949	Down	NELFB (-3315)
LOC100507387	NR_038402	chr5 : 175546551 - 175552168	Down	THOC3 (-154058), SIMC1 (-116005)
LOC100506746	NR_038841	chr4 : 87846045 - 87856002	Down	SLC10A6 (-80608), AFF1 (-5130)
LINC00672	NR_038847	chr17 : 37081420 - 37085637	Down	LASP1 (+57417), PLXDC1 (+224373)
LINC00311	NR_038859	chr16 : 85316563 - 85321685	Down	GSE1 (-327698), KIAA0513 (+222306)
LINC01268	NR_038863	chr6 : 114189178 - 114194512	Down	MARCKS (+13304), HDAC2 (+100604)
LINC01426	NR_038885	chr21 : 36118121 - 36157168	Down	CLIC6 (+95957), RUNX1 (+283996)
LOC401320	NR_038889	chr7 : 30587972 - 30617395	Down	GGCT (-58224), GARS (-31613)
LINC00641	NR_038970	chr14 : 21668237 - 21675059	Down	OR5AU1 (-47426), HNRNPC (+65990)
RBM26-AS1	NR_038991	chr13 : 79980443 - 79998468	Down	NDFIP2 (-65831), RBM26 (-9533)
LOC286437	NR_039980	chrX : 103139054 - 103401708	Down	H2BFWT (-2122)
LINC01004	NR_039981	chr7 : 104622193 - 104631612	Down	KMT2E (-27723), LHFPL3 (+657799)
LOC100130899	NR_039988	chr22 : 40428335 - 40432581	Down	TNRC6B (-143471), GRAP2 (+133372)
LINC01569	NR_039999	chr16 : 4295825 - 4303790	Down	SRL (-7727), TFAP4 (+23268)
ERVK13-1	NR_040023	chr16 : 2708389 - 2723440	Down	KCTD5 (-16561), PDPK1 (+127950)
LOC157273	NR_040039	chr8 : 9182560 - 9192590	Down	TNKS (-225849), PPP1R3B (-179369)
LOC100287015	NR_040040	chr8 : 6261076 - 6264069	Down	MCPH1 (-1540)

RAD51-AS1	NR_040058	chr15 : 40985952 - 40987303	Down	RAD51 (-744)
SMG7-AS1	NR_040063	chr1 : 183430010 - 183441117	Down	NMNAT2 (-47827), SMG7 (-6074)
LOC100506990	NR_040091	chr8 : 12294521 - 12523120	Down	DEFB130 (-232996), LONRF1 (+204178)
ENTPD3-AS1	NR_040100	chr3 : 40428672 - 40494799	Down	RPL14 (-37095), ENTPD3 (+33089)
LOC728175	NR_040108	chr4 : 185262183 - 185275130	Down	ENPP6 (-129543), IRF2 (+127077)
PCAT19	NR_040109	chr19 : 41960073 - 42006554	Down	CEACAM21 (-99287), ATP5SL (-37870)
SNAP25-AS1	NR_040710	chr20 : 10004459 - 10288066	Down	SNAP25 (-53215), ANKEF1 (+130566)
GAS6-AS1	NR_044995	chr13 : 114518582 - 114567046	Down	GAS6 (+24226), TMEM255B (+80598)
PRICKLE2-AS1	NR_045697	chr3 : 64053639 - 64211131	Down	PSMD6 (-123149), PRICKLE2 (+78746)
LINC00864	NR_046091	chr10 : 89156330 - 89167457	Down	GLUD1 (-307271), MINPP1 (-102738)
LINC01146	NR_046094	chr14 : 88490893 - 88553688	Down	GPR65 (+50823), KCNK10 (+267302)
LOC284581	NR_046097	chr1 : 205831206 - 205865215	Down	PM20D1 (-28966), SLC26A9 (+64377)
MIRLET7DHG	NR_046163	chr9 : 96938851 - 96966848	Down	ZNF169 (-68743), PTPDC1 (+106104)
MGC27345	NR_046216	chr7 : 127937737 - 127947816	Down	RBM28 (+41185), LEP (+61440)
NIPBL-AS1	NR_046262	chr5 : 36871462 - 36876796	Down	NIPBL (-2732)
LOC100130744	NR_046285	chr5 : 14704908 - 14871887	Down	ANKH (+83489), FAM105B (+123541)
LOC171391	NR_046316	chr11 : 779453 - 780755	Down	PDDC1 (-2620)
LOC728040	NR_046377	chr4 : 74374519 - 74394250	Down	AFM (+36985), RASSF6 (+101963)
PRICKLE2-AS3	NR_046702	chr3 : 64053639 - 64211131	Down	PSMD6 (-123149), PRICKLE2 (+78746)
LINC00854	NR_047479	chr17 : 41373436 - 41381062	Down	ARL4D (-99078), TMEM106A (+13355)
LOC729732	NR_047662	chr8 : 12294521 - 12523120	Down	DEFB130 (-232996), LONRF1 (+204178)
UGDH-AS1	NR_047679	chr4 : 39529458 - 39640481	Down	UGDH (-55732), SMIM14 (+55740)
ASB16-AS1	NR_049729	chr17 : 42248073 - 42264085	Down	TMUB2 (-8338), ASB16 (+8005)
PTCSC3	NR_049735	chr14 : 36539632 - 36645857	Down	MBIP (+197137), BRMS1L (+297221)
TMEM9B-AS1	NR_073431	chr11 : 8986221 - 8997830	Down	TMEM9B (-5706), NRIP3 (+33570)
TMEM220-AS1	NR_073455	chr17 : 10616638 - 10718481	Down	TMEM220 (-33927), PIRT (+74202)
LOC100506688	NM_001242737	chr5 : 988424 - 997455	Down	BRD9 (-100001), NKD2 (-16004)
LINC01750	NR_145438.1	chr1 : 112533189 - 112541463	Down	CTTNBP2NL (-401477), KCND3 (-5549)

Plus (+) means upstream while minus (-) means downstream.

Two genes omitted since chromosome location not well-established.

Table S6. Biological processes assigned using GO-slim annotation (100 µM DEET).

No.	Biological Process (with corresponding GO[a] ID)	Hits
1	cellular process (GO:0009987)	14
2	metabolic process (GO:0008152)	13
3	localization (GO:0051179)	5
4	response to stimulus (GO:0050896)	5
5	immune system process (GO:0002376)	4
6	biological regulation (GO:0065007)	3
7	developmental process (GO:0032502)	2
8	multicellular organismal process (GO:0032501)	2
9	cell killing (GO:0001906)	2
10	cellular component organization or biogenesis (GO:0071840)	1
11	locomotion (GO:0040011)	1

[a](GO) is Gene Ontology identification number.

Table S7. Biological processes assigned using GO-slim annotation (10 µM Fipronil).

No.	Biological Process (with corresponding GO[a] ID)	Hits
1	cellular process (GO:0009987)	192
2	metabolic process (GO:0008152)	185
3	response to stimulus (GO:0050896)	53
4	localization (GO:0051179)	44
5	biological regulation (GO:0065007)	41
6	developmental process (GO:0032502)	41
7	multicellular organismal process (GO:0032501)	37
8	cellular component organization or biogenesis (GO:0071840)	29
9	immune system process (GO:0002376)	29
10	reproduction (GO:0000003)	8
11	biological adhesion (GO:0022610)	7
12	locomotion (GO:0040011)	3
13	cell killing (GO:0001906)	2

14	rhythmic process (GO:0048511)	1

[a](GO) is Gene Ontology identification number.

Table S8. Biological processes assigned using GO-slim annotation (100 µM DEET+ 10 µM Fipronil).

No.	Biological Process (with corresponding GO[a] ID)	Hits
1	cellular process (GO:0009987)	223
2	metabolic process (GO:0008152)	205
3	response to stimulus (GO:0050896)	67
4	localization (GO:0051179)	59
5	developmental process (GO:0032502)	53
6	biological regulation (GO:0065007)	49
7	immune system process (GO:0002376)	45
8	multicellular organismal process (GO:0032501)	38
9	cellular component organization or biogenesis (GO:0071840)	37
10	biological adhesion (GO:0022610)	11
11	reproduction (GO:0000003)	9
12	locomotion (GO:0040011)	5
13	rhythmic process (GO:0048511)	2
14	cell killing (GO:0001906)	2

[a](GO) is Gene Ontology identification number.

Table S9. Signaling pathways assigned using PANTHER (10 µM Fipronil).

No.	Signaling Pathway (with corresponding PANTHER[a] ID)	Hits
1	Ubiquitin proteasome pathway (P00060)	7
2	Gonadotropin-releasing hormone receptor pathway (P06664)	6
3	Angiogenesis (P00005)	5
4	Wnt signaling pathway (P00057)	5
5	T cell activation (P00053)	5
6	Integrin signaling pathway (P00034)	4
7	Heterotrimeric G-protein signaling pathway-Gq alpha and Go alpha mediated pathway (P00027)	4
8	TGF-beta signaling pathway (P00052)	4

9	Apoptosis signaling pathway (P00006)	3
10	Interleukin signaling pathway (P00036)	3
11	Insulin/IGF pathway-protein kinase B signaling cascade (P00033)	3
12	Inflammation mediated by chemokine and cytokine signaling pathway (P00031)	3
13	p53 pathway (P00059)	3
14	Heterotrimeric G-protein signaling pathway-Gi alpha and Gs alpha mediated pathway (P00026)	3
15	Ras Pathway (P04393)	3
16	EGF receptor signaling pathway (P00018)	3
17	Parkinson disease (P00049)	3
18	Notch signaling pathway (P00045)	3
19	CCKR signaling map (P06959)	3
20	Huntington disease (P00029)	2
21	p53 pathway feedback loops 2 (P04398)	2
22	VEGF signaling pathway (P00056)	2
23	FGF signaling pathway (P00021)	2
24	P53 pathway feedback loops 1 (P04392)	2
25	PDGF signaling pathway (P00047)	2
26	Nicotinic acetylcholine receptor signaling pathway (P00044)	2
27	De novo purine biosynthesis (P02738)	1
28	Adrenaline and noradrenaline biosynthesis (P00001)	1
29	Insulin/IGF pathway-mitogen activated protein kinase kinase/MAP kinase cascade (P00032)	1
30	Hypoxia response via HIF activation (P00030)	1
31	Asparagine and aspartate biosynthesis (P02730)	1
32	Nicotine pharmacodynamics pathway (P06587)	1
33	p53 pathway by glucose deprivation (P04397)	1
34	Thyrotropin-releasing hormone receptor signaling pathway (P04394)	1
35	Toll receptor signaling pathway (P00054)	1
36	General transcription by RNA polymerase I (P00022)	1
37	Bupropion degradation (P05729)	1
38	PI3 kinase pathway (P00048)	1
39	Opioid proopiomelanocortin pathway (P05917)	1
40	Nicotine degradation (P05914)	1

41	Glutamine glutamate conversion (P02745)	1
42	Cadherin signaling pathway (P00012)	1
43	Dopamine receptor mediated signaling pathway (P05912)	1
44	B cell activation (P00010)	1
45	Cortocotropin releasing factor receptor signaling pathway (P04380)	1

[a]PANTHER (**P**rotein **AN**alysis **TH**rough **E**volutionary **R**elationships) identification number.

Table S10. Signaling pathways assigned using PANTHER (100 µM DEET+ 10 µM Fipronil).

No.	Signaling Pathway (with corresponding PANTHER[a] ID)	Hits
1	Heterotrimeric G-protein signaling pathway-Gi alpha and Gs alpha mediated pathway (P00026)	7
2	T cell activation (P00053)	7
3	Gonadotropin-releasing hormone receptor pathway (P06664)	7
4	Ubiquitin proteasome pathway (P00060)	6
5	Angiogenesis (P00005)	5
6	Integrin signaling pathway (P00034)	5
7	Inflammation mediated by chemokine and cytokine signaling pathway (P00031)	5
8	p53 pathway (P00059)	5
9	Heterotrimeric G-protein signaling pathway-Gq alpha and Go alpha mediated pathway (P00027)	5
10	Wnt signaling pathway (P00057)	5
11	Insulin/IGF pathway-protein kinase B signaling cascade (P00033)	4
12	Ras Pathway (P04393)	4
13	TGF-beta signaling pathway (P00052)	4
14	EGF receptor signaling pathway (P00018)	4
15	PDGF signaling pathway (P00047)	4
16	Nicotinic acetylcholine receptor signaling pathway (P00044)	4
17	CCKR signaling map (P06959)	4
18	Apoptosis signaling pathway (P00006)	3
19	p53 pathway feedback loops 2 (P04398)	3
20	Parkinson disease (P00049)	3
21	Notch signaling pathway (P00045)	3
22	Cortocotropin releasing factor receptor signaling pathway (P04380)	3
23	Interleukin signaling pathway (P00036)	2

24	Alzheimer disease-presenilin pathway (P00004)	2
25	Alzheimer disease-amyloid secretase pathway (P00003)	2
26	Alpha adrenergic receptor signaling pathway (P00002)	2
27	Phenylethylamine degradation (P02766)	2
28	Adrenaline and noradrenaline biosynthesis (P00001)	2
29	Hypoxia response via HIF activation (P00030)	2
30	Huntington disease (P00029)	2
31	VEGF signaling pathway (P00056)	2
32	Thyrotropin-releasing hormone receptor signaling pathway (P04394)	2
33	P53 pathway feedback loops 1 (P04392)	2
34	Endothelin signaling pathway (P00019)	2
35	PI3 kinase pathway (P00048)	2
36	Opioid proopiomelanocortin pathway (P05917)	2
37	Cadherin signaling pathway (P00012)	2
38	Dopamine receptor mediated signaling pathway (P05912)	2
39	Beta3 adrenergic receptor signaling pathway (P04379)	1
40	Metabotropic glutamate receptor group III pathway (P00039)	1
41	Beta2 adrenergic receptor signaling pathway (P04378)	1
42	Beta1 adrenergic receptor signaling pathway (P04377)	1
43	Ionotropic glutamate receptor pathway (P00037)	1
44	5HT4 type receptor mediated signaling pathway (P04376)	1
45	De novo purine biosynthesis (P02738)	1
46	5HT3 type receptor mediated signaling pathway (P04375)	1
47	5HT2 type receptor mediated signaling pathway (P04374)	1
48	5HT1 type receptor mediated signaling pathway (P04373)	1
49	Insulin/IGF pathway-mitogen activated protein kinase kinase/MAP kinase cascade (P00032)	1
50	Asparagine and aspartate biosynthesis (P02730)	1
51	Nicotine pharmacodynamics pathway (P06587)	1
52	Synaptic vesicle trafficking (P05734)	1
53	GABA-B receptor II signaling (P05731)	1
54	p53 pathway by glucose deprivation (P04397)	1
55	Toll receptor signaling pathway (P00054)	1

56	FGF signaling pathway (P00021)	1
57	Oxytocin receptor mediated signaling pathway (P04391)	1
58	Bupropion degradation (P05729)	1
59	Opioid prodynorphin pathway (P05916)	1
60	Opioid proenkephalin pathway (P05915)	1
61	Nicotine degradation (P05914)	1
62	Glutamine glutamate conversion (P02745)	1
63	Muscarinic acetylcholine receptor 2 and 4 signaling pathway (P00043)	1
64	Blood coagulation (P00011)	1
65	Muscarinic acetylcholine receptor 1 and 3 signaling pathway (P00042)	1
66	B cell activation (P00010)	1
67	Metabotropic glutamate receptor group II pathway (P00040)	1
68	Pyrimidine Metabolism (P02771)	1

[a]PANTHER (**P**rotein **AN**alysis **TH**rough **E**volutionary **R**elationships) identification number.

S1. Chromosomal Distribution of Dysregulated lncRNAs

Table S1 shows the distribution of lncRNAs whose transcripts were differentially expressed ($P \leq 0.01$) in primary human hepatocytes for each chromosome. In the 100 μM DEET treatment, chromosome 16 had the most lncRNAs affected (three) while only two lncRNAs were dysregulated on each of chromosomes 5, 6, 7, 9, 11, and 19. Since there were only 20 lncRNAs in total affected by the DEET-only treatment, the rest of the chromosomes only had one or no lncRNAs dysregulated by the treatment. There were many more lncRNAs whose transcripts were up- or downregulated by the 10 μM fipronil treatment (269 total or 13.5X as many), which led to all 23 of the chromosomes having lncRNAs that were affected by the treatment. The three chromosomes with the most lncRNAs affected were chromosomes 1, 7, and 10, while the chromosomes with the lowest number of lncRNAs affected were chromosomes 13 and 18. For the mixture of 100 μM DEET and 10 μM fipronil treatments, the three chromosomes that had the most lncRNAs dysregulated by the treatment were chromosomes 1, 6, and 7 while chromosomes 13, 18, and 21 were the three with the lowest number of affected lncRNAs.

Examining the percentage of lncRNAs dysregulated on each chromosome (up- and downregulated combined) compared to the total number of genes for each chromosome (using the latest Ensembl release [25]), showed for the 100 μM DEET treatment, chromosome 16 had 0.13% of its genes affected by the treatment (3 out of 2,375), which was the highest percentage of genes affected across all chromosomes for this treatment. The next closest was chromosome 9, where 0.09% of the genes were dysregulated (2 out of 2,224) and chromosomes 2, 3, 8, 12, 13, 14, 15, 18, 20, 21, and 22 had no lncRNAs that were affected in response to 100 μM DEET. The three chromosomes most affected by the 10 μM fipronil treatment were chromosome 7 (0.78%), chromosome 10 (0.78%), and chromosome 9 (0.67%) while the three chromosomes least affected were chromosome 13 (0.16%), chromosome 14 (0.23%), and chromosome 17 (0.28%). In the 100 μM DEET plus 10 μM fipronil mixture, the three most affected chromosomes were chromosome 7 (1.02%), chromosome 16 (0.88%), and chromosome 10 (0.87%) and the three least affected were chromosome 13 (0.24%), chromosome 8 (0.30%), and chromosome X (0.34%).

S2. Comparison of Dysregulated lncRNA and Protein-Coding Gene Chromosomal Distribution

We compared our findings on the chromosomal distribution of lncRNA genes with up- and downregulated transcripts to the chromosomal distribution of protein-coding genes dysregulated after primary human hepatocytes were exposed to both DEET and fipronil using the percentage of genes dysregulated versus the total number of genes on each chromosome. Table S2 shows the chromosomal distribution of protein-coding genes significantly dysregulated ($P \leq 0.01$) after primary human hepatocytes were treated with 100 μM DEET, 10 μM fipronil, and a mixture of 100 μM DEET plus 10 μM fipronil. It takes into account the most recent Ensembl gene annotations from December of 2016 [25] in calculations comparing the number of dysregulated protein-coding genes to the total number of coding and noncoding genes on each chromosome. For the 100 μM DEET treatment the three chromosomes that had the most dysregulated protein-coding genes were chromosome 7 (0.57%), chromosome 1 (0.50%), and chromosome 4 (0.57%). These percentages did not correspond with the top three genes that had the most lncRNAs affected in the DEET-only treatment. In the 10 μM fipronil treatment, the three chromosomes with the most dysregulated protein-coding genes were chromosome 1 (7.92%), chromosome 12 (7.53%), and chromosome 16 (7.49%) and the three chromosomes with the lowest number of dysregulated protein-coding genes were chromosome 13 (3.84%), chromosome 14 (4.72%), and chromosome 18 (4.73%). Chromosomes 13 and 14 had the least number of lncRNAs with differentially expressed transcripts in the 10 μM fipronil treatment and no lncRNAs dysregulated in the 100 μM DEET treatment, which suggests that these two chromosomes play a smaller role in the response of liver cells to DEET and fipronil at these concentrations than the other chromosomes. There was little correlation between the chromosomes with

the most dysregulated lncRNAs and protein-coding genes in response to 10 μM fipronil. The three chromosomes with the highest number of differentially expressed transcripts from protein-coding genes in response to the mixture of 100 μM DEET plus 10 μM fipronil were chromosome 19 (12.27%), chromosome 1 (10.99%), and chromosome 12 (10.21%). There was no obvious correlation with the dysregulated lncRNA profile and the protein-coding gene profile in response to the mixture either. Chromosome 13 (as it did in the 100 μM DEET and 10 μM fipronil treatments) had the lowest percentage of protein-coding genes affected by the 100 μM DEET plus 10 μM fipronil treatment (5.57%), suggesting that chromosome 13 is less important in the human hepatocyte response to a mixture of DEET and fipronil than any other chromosomes if we base our assumption on the percent of dysregulated genes versus the total number of genes per chromosome.

In summary, 20 lncRNA genes in primary human hepatocytes had transcripts that were significantly up- or downregulated ($P \leq 0.01$) by 100 μM DEET, 269 by 10 μM fipronil (13.5X the number dysregulated by 100 μM DEET only), and 333 by a mixture of 100 μM DEET and 10 μM fipronil (1.2X the number dysregulated by 10 μM fipronil alone and 16.7X the number dysregulated by 100 μM DEET alone). Therefore, 0.04% of the total number of known genes (both coding and noncoding) were lncRNAs dysregulated by exposure to 100 μM DEET, 0.48% were lncRNAs affected by the 10 μM fipronil treatment, and 0.59% were lncRNAs dysregulated by the 100 μM DEET plus 10 μM fipronil treatment. We observed a more-than-additive effect with the mixture of 100 μM DEET and 10 μM fipronil together (333 lncRNAs whose transcripts were up- or downregulated) versus a purely additive effect that would have totaled 289 lncRNAs with differentially expressed transcripts, which was the sum of the dysregulated lncRNAs from the 100 μM DEET treatment and the 10 μM fipronil treatment. This reveals that more lncRNA transcripts, and protein-coding transcripts from our previous study [18], were up- or downregulated in primary human hepatocytes to metabolize a mixture of 100 μM DEET and 10 μM fipronil together than either chemical alone. Interestingly, the concentration of fipronil used was 10-fold lower than the concentration of DEET used (10 μM fipronil versus 100 μM DEET) suggesting that a 100 μM fipronil dosage might elicit a much stronger response than a 100 μM DEET dosage. These findings underline the importance of studying environmental chemicals as they are more typically encountered, which is in combination rather than singly, especially to vulnerable populations like agricultural field workers, for example, that may encounter these substances more regularly.

S3. GREAT Analysis of LncRNA-Coding Gene Relationships

Figure S1A-C displays the number of neighboring protein-coding genes per dysregulated input lncRNA sequence, the absolute distance to the closest TSS in kilobases (kb) for each dysregulated lncRNA transcription site, and the orientation and distance of each dysregulated lncRNA gene to its closest TSS, respectively, after 100 μM DEET exposure. Some of the protein-coding genes with differentially expressed transcripts that we identified previously and lay within 1000 kb of these 20 dysregulated lncRNA genes were not designated as targets in the GREAT analysis, but we chose to include them in all further downstream analysis. In Figure S1A, only 4 dysregulated lncRNAs had one neighboring protein-coding gene while 16 of the dysregulated lncRNAs had two neighboring protein-coding genes within 1000 kb. Figure S1B shows that 2 lncRNAs were within 0–5 kb of their closest protein-coding gene TSS, 14 lncRNAs were within 5–50 kb, 18 were within 50-500 kb, and 2 were 500–1000 kb away. This implies that in previous gene-based tools focused on this type of analysis, over half of our dataset would have been excluded based on distance to the nearest TSS. Figure S1C shows the distance and orientation of the 20 dysregulated lncRNAs versus their closest neighboring protein-coding genes, where 23 were upstream of the TSS and 13 were downstream. Similar bar graphs are displayed for the 10 μM fipronil treatment (Figure S1D-F) and the 100 μM DEET plus 10 μM fipronil mixture (Figure S1G-I).

S4. Well-Studied LncRNAs Dysregulated by DEET and Fipronil Treatments

S4.1 Highly up-regulated in liver cancer (HULC) transcripts down-regulated

HULC transcripts were significantly down-regulated in the 10 μM fipronil treatment (log2FC of -0.57). While *HULC* expression is known to influence the development of hepatocellular carcinoma (HCC), the most common type of liver cancer, there are conflicting reports regarding its over or under-expression in relation to HCC [66]. In pancreatic cancer *HULC* overexpression is positively correlated with larger tumors and decreased survivability [67] and in gastric cancer *HULC* overexpression contributed to lymph node metastasis [68]. However, liver studies by Yang et al (2015) established that higher levels of HULC in HCC resulted in less vascular invasion and increased survivability in some instances, which conflicts with other HCC studies [69]. It is also known that HULC can act as a miRNA sponge to reduce miRNA activity [70]. One miRNA type that HULC interacts with is mir-372, which is known to suppress tumorigenesis in certain types of cancer like endometrial carcinoma. The up-regulation of *HULC* could therefore potentially inhibit the tumor suppression capability of mir-372 [71]. Wu et al. (2015) demonstrated that down-regulation of mir-372 was correlated with tumor metastasis and poor prognosis in HCC [72]. Therefore, downregulation of *HULC* may also be a positive in certain situations where an abundance of mir-372 may be necessary to combat HCC. Focused research is needed to determine in what scenarios the up- or downregulation of *HULC* can serve as a prognostic or diagnostic indicator of liver disease, which is likely to differ between stages and types.

S4.2. H19 transcripts up-regulated

The *H19* gene, sometimes referred to as long intergenic non-protein coding RNA 8, codes for an lncRNA whose transcripts were up-regulated in both the 10 μM fipronil and 100 μM DEET plus 10 μM fipronil treatments (log2FC of +1.55 and +0.59, respectively). Overexpression of H19 is associated with tumorigenesis in several different tissue types, and blocking H19 in breast cancer and HCC cells reduced their ability to grow and develop [73,74]. H19 also has the ability to affect p53 (discussed in more detail later) which has been called the "master regulator" due to its ability to prevent genome mutations. H19 was up-regulated in cells containing mutant p53 where oxygen conditions were low, but its expression remained normal when oxygen levels were sufficient [75].

S4.3. Metastasis associated lung adenocarcinoma transcript 1 (MALAT1) transcripts down-regulated

MALAT1 transcripts were significantly downregulated in the 100 μM DEET, 10 μM fipronil, and 100 μM DEET plus 10 μM fipronil treatments (log2FC of -1.34, -1.87, and -2.28 respectively) and is one of the most studied lncRNAs. It plays a multitude of roles in processes including gene splicing and nuclear organization, but its overexpression is related to several types of cancer. *MALAT1* overexpression promotes malignancy in cancer cells and controls gene expression of several metastasis-associated transcripts in lung cancer cells [76]. However, the down-regulation of MALAT1 (as we observed in primary human hepatocytes) was shown to induce tumor progression in a recent breast cancer study [77]. Therefore the over- or under-expression of MALAT1 is likely very tissue specific. Downregulation of MALAT1 as we observed in our data set could play a completely different role in primary liver cells than it does in other tissues and systems. We must consider that many of the studies determining the role of MALAT1 were conducted in immortalized cell lines or model non-human organisms and not primary human hepatocytes [68].

S4.4. Nuclear enriched abundant transcript 1 (NEAT1) transcripts down-regulated

NEAT1 transcripts were also downregulated in all three of our treatment conditions (log2FC of -1.10 for 100 μM DEET, -1.22 for 10 μM fipronil, and -1.52 for the 100 μM DEET plus 10 μM fipronil mixture). The lncRNA NEAT1 plays an important role in the formation of nuclear paraspeckles, which are sub-nuclear bodies formed in response to stress, viral infection, and circadian rhythm maintenance. NEAT1 is more of an architectural components that interacts with proteins that have a direct role in transcription and RNA processing. Downregulation of NEAT1 results in impairment or inhibition of paraspeckle formation that could have a profound effect on some of the processes already mentioned above like response to viral infection [78]. Interestingly, recent studies demonstrate that NEAT1 and MALAT1 co-localize to hundreds of genomic sites likely due to cues from the transcription process and not specific DNA sequences [79]. Therefore, disruption of either of these lncRNAs could affect many signaling pathways in a positive or negative manner either by their interaction with one another or on genetic components that they both may influence.

S4.5. X-inactive specific transcript (XIST) and TSIX transcripts down-regulated

X-inactive specific transcript (XIST) and TSIX transcripts were both significantly downregulated by the 10 μM fipronil and 100 μM DEET plus 10 μM fipronil treatments (XIST, log2FC of -0.60 and -0.93, respectively; TSIX, log2FC of -0.59 and -0.97, respectively). Both XIST and TSIX are lncRNAs involved in the process of X inactivation where certain genes on the sex chromosomes are repressed to equalize gene expression among the sexes. The function of XIST is to coat certain regions of the sex chromosomes and suppress gene expression at the coating site (i.e., X inactivation) while TSIX, the antisense of XIST, functions to downregulate the expression of XIST when necessary [80]. It would seem logical that the repression of both of these lncRNAs would disrupt functionality of the entire X inactivation system but further study is required.

S4.6. Maternally expressed 3 (MEG3) transcripts down-regulated

MEG3 transcripts were downregulated in both the 10 μM fipronil and 100 μM DEET plus 10 μM fipronil treatments (log2FC of -1.19 and -1.70, respectively). This lncRNA activates p53 and functions as a tumor suppressor, so its expression is typically reduced or lost in cancer cells. A 2015 study revealed that MEG3 interacts with several TGF-β pathway genes, which are important in several cellular processes like cell growth, differentiation, and apoptosis [81,82]. Therefore, dysregulation of MEG3 could negatively influence all of these processes.

Reference

66. Yu, X.; Zheng, H.; Chan, M.T.; Wu, W.K.K. Hulc: An oncogenic long non-coding RNA in human cancer. *J. Cell. Mol. Med.* **2016**, *21*, 410–417.
67. Peng, W.; Gao, W.; Feng, J. Long noncoding RNA hulc is a novel biomarker of poor prognosis in patients with pancreatic cancer. *Med. Oncol.* **2014**, *31*, 1–7.
68. Zhao, Y.; Guo, Q.; Chen, J.; Hu, J.; Wang, S.; Sun, Y. Role of long non-coding RNA hulc in cell proliferation, apoptosis and tumor metastasis of gastric cancer: A clinical and in vitro investigation. *Oncol. Rep.* **2014**, *31*, 358–364.
69. Yang, Z.; Lu, Y.; Xu, Q.; Tang, B.; Park, C.-K.; Chen, X. HULC and H19 played different roles in overall and disease-free survival from hepatocellular carcinoma after curative hepatectomy: A preliminary analysis from gene expression omnibus. *Dis. Markers* **2015**, *2015*, 191029.

70. Li, C.H.; Chen, Y. Targeting long non-coding RNAs in cancers: Progress and prospects. *Int. J. Biochem. Cell Biol.* **2013**, *45*, 1895–1910.
71. Liu, B.-L.; Sun, K.-X.; Zong, Z.-H.; Chen, S.; Zhao, Y. MicroRNA-372 inhibits endometrial carcinoma development by targeting the expression of the ras homolog gene family member c (RHOC). *Oncotarget* **2016**, *7*, 6649.
72. Wu, G.; Wang, Y.; Lu, X.; He, H.; Liu, H.; Meng, X.; Xia, S.; Zheng, K.; Liu, B. Low mir-372 expression correlates with poor prognosis and tumor metastasis in hepatocellular carcinoma. *BMC Cancer* **2015**, *15*, 182.
73. Barsyte-Lovejoy, D.; Lau, S.K.; Boutros, P.C.; Khosravi, F.; Jurisica, I.; Andrulis, I.L.; Tsao, M.S.; Penn, L.Z. The c-myc oncogene directly induces the h19 noncoding RNA by allele-specific binding to potentiate tumorigenesis. *Cancer Res.* **2006**, *66*, 5330–5337.
74. Berteaux, N.; Lottin, S.; Monté, D.; Pinte, S.; Quatannens, B.; Coll, J.; Hondermarck, H.; Curgy, J.-J.; Dugimont, T.; Adriaenssens, E. H19 mRNA-like noncoding RNA promotes breast cancer cell proliferation through positive control by E2F1. *J. Biol. Chem.* **2005**, *280*, 29625–29636.
75. Matouk, I.J.; Mezan, S.; Mizrahi, A.; Ohana, P.; Abu-lail, R.; Fellig, Y.; Galun, E.; Hochberg, A. The oncofetal h19 RNA connection: Hypoxia, p53 and cancer. *BBA Mol. Cell Res.* **2010**, *1803*, 443–451.
76. Gutschner, T.; Hämmerle, M.; Eissmann, M.; Hsu, J.; Kim, Y.; Hung, G.; Revenko, A.; Arun, G.; Stentrup, M.; Gross, M. The noncoding RNA malat1 is a critical regulator of the metastasis phenotype of lung cancer cells. *Cancer Res.* **2013**, *73*, 1180–1189.
77. Yang, Z.; Lu, W.; Ning, L.; Hao, D.; Jian, S.; Hai-Feng, C. Downregulation of long non-coding RNA malat1 induces tumor progression of human breast cancer through regulating CCND1 expression. *Open Life Sci.* **2016**, *11*, 232–236.
78. Fox, A.H.; Lamond, A.I. Paraspeckles. *Cold Spring Harb. Perspect. Biol.* **2010**, *2*, a000687.
79. West, J.A.; Davis, C.P.; Sunwoo, H.; Simon, M.D.; Sadreyev, R.I.; Wang, P.I.; Tolstorukov, M.Y.; Kingston, R.E. The long noncoding RNAs neat1 and malat1 bind active chromatin sites. *Mol. Cell* **2014**, *55*, 791–802.
80. Lee, J.; Davidow, L.S.; Warshawsky, D. Tsix, a gene antisense to xist at the x-inactivation centre. *Nat. Genet.* **1999**, *21*, 400–404.
81. Markowitz, S.D.; Roberts, A.B. Tumor suppressor activity of the TGF-β pathway in human cancers. *Cytokine Growth Factor Rev.* **1996**, *7*, 93–102.
82. Mondal, T.; Subhash, S.; Vaid, R.; Enroth, S.; Uday, S.; Reinius, B.; Mitra, S.; Mohammed, A.; James, A.R.; Hoberg, E. Meg3 long noncoding RNA regulates the TGF-β pathway genes through formation of RNA-DNA triplex structures. *Nat. Commun.* **2015**, *6*, 7743.
